# Mutated sigma-1R disrupts cell homeostasis in dHMN patient cells

**DOI:** 10.1007/s00018-025-05676-y

**Published:** 2025-04-09

**Authors:** Sofia Zanin, Francesco Ciscato, Antonio Petrucci, Annalisa Botta, Federico Chiossi, Giovanni Vazza, Rosario Rizzuto, Giorgia Pallafacchina

**Affiliations:** 1 Laboratory for Genetics of Mitochondrial Disorders, UMR 1163, Institut Imagine, Université de Paris, Paris, France; 2 Neuroscience Institute, Italian National Research Council CNR, Padua, Italy; 3 Department of Biomedical Sciences, University of Padua, Padua, Italy; 4 Center for Neuromuscular and Neurological Rare Diseases, S. Camillo Forlanini Hospital, Rome, Italy; 5 Medical Genetics Section, Department of Biomedicine and Prevention, University of Rome Tor Vergata, Rome, Italy; 6 Institut de Recherche de Chimie, Chimie ParisTech, PSL University, CNRS, Paris, France; 7 Department of Biology, University of Padua, Padua, Italy

**Keywords:** Sigma-1R, Hereditary neuropathies, Ca^2+^ signalling, ER-mitochondria contacts, Cellular proteostasis

## Abstract

Hereditary-Motor-Neuropathies (dHMNs) are clinically and genetically heterogeneous neurological disorders characterized by degeneration of peripheral motoneurons. We previously identified two sigma-1 receptor (Sigma-1R) variants (p.E138Q; p.E150K) in dHMN Italian patients that behave as “loss-of-function” mutations in neuroblastoma cell lines. Here, we characterize the functional effects of Sigma-1R mutation in primary fibroblasts from homozygous patients bearing the E150K mutation, and matched controls, by performing biochemical, gene expression, immunofluorescence and Ca^2+^ imaging analysis. Our results show that Sigma-1R expression and distribution is significantly altered in patient fibroblasts. Moreover, patient cells present a general derangement of cell homeostasis as revealed by impairment of global Ca^2+^ dynamics, disorganization of the ER-mitochondria tethers, enhancement of the autophago-lysosomal pathway and blunting of mitochondrial aerobic metabolism compared to controls. These findings highlight the crucial role of Sigma-1R in the maintenance of cell and protein homeostasis, inter-organelle communication and intracellular Ca^2+^ signalling, supporting the notion that Sigma-1R is protective for motor neuron activity and its down-regulation and/or loss-of-function, as in the case of the E150K mutation, might play the key role in the neuronal degeneration in dHMN patients.

**Supplementary Information:**

The online version contains supplementary material available at 10.1007/s00018-025-05676-y.

## Introduction

Sigma-1 receptor protein (Sigma-1R) is a ubiquitously expressed protein of the endoplasmic reticulum (ER) membranes, with chaperone function, which has been shown to regulate multiple cellular responses, from the ER-Ca^2+^ release, through the binding and modulation of IP_3_R activity [1, 2], to the control of plasma membrane ion channels [3–6] via direct interaction or interaction with channel regulators, such as Stim1 [7], as well as modulation of G-protein coupled receptors [8]. The Sigma-1R gene, *SIGMAR1,* was originally cloned in 1996 [9] and it encodes a 223 amino acid, ~ 25 KDa, single transmembrane helical integral protein [10], which resides at specialized regions of the ER in close contact with mitochondria called mitochondria-associated membranes or MAMs [1]. The latest data, indicated that Sigma-1R globular C-terminal ligand-binding domain faces the ER lumen, while its short N-terminus is on the cytosolic side [11]. This confuted the previous topological model of Sigma-1R based on crystallographic data, according to which the C-terminal globular domain faced the cytosol [10].

Sigma-1R protein is extremely abundant in the nervous system where it has been shown to exert a neuromodulatory and neuroprotective action, which ranges from increasing synaptic stability to combating oxidative stress and promoting cell survival in different neuronal cell types [12–15]. In addition, Sigma-1 R is involved in numerous neurologic and psychiatric conditions, memory deficit and neurodegenerative pathologies like amyotrophic lateral sclerosis (ALS), Alzheimer and Parkinson’s Disease [16–22], for a more exhaustive review see [23]. Moreover, its deletion and down regulation have been associated with exacerbation of the pathological phenotype in a number of neurodegenerative conditions [24–26] and, very recently, the use of Sigma-1R ligands was realized in clinics [27]. Finally, several studies, including that from our group, associated Sigma-1R mutations with hereditary neuropathies of the distal motor neurons [28–35].

Distal hereditary motor neuropathies (dHMNs) belong to a heterogeneous group of inherited motor disorders of the peripheral nervous system. Patients affected by dHMN progressively manifest distal limb muscle weakness and atrophy without major sensory involvement. To date, more than 30 genes have been reported as causative of dHMN, nevertheless, more than 70% of cases these diseases remain without a genetic explanation [36, 37]. dHMN-linked genes encode proteins implicated in different cellular pathways including protein synthesis, transcription regulation, axonal trafficking, cytoskeleton stability and ER dynamics [36, 37], supporting the notion that different pathogenic mechanisms may lead to a common phenotype characterized by motor neuron degeneration, which is the hallmark of this group of diseases*.*

In 2016, we identified *SIGMAR1* as the causative gene for a recessive form of dHMN in two Italian families [30], showing that the overexpression of Sigma-1R dHMN variants in neuronal cells affects the intracellular organelle communication and Ca^2+^ signalling and, at the same time, dramatically enhanced autophagosome formation. The involvement of Sigma-1R in the promotion of cell survival and homeostasis of Ca^2+^ signals have been supported by knock-down experiments or expression of different pathologic Sigma-1R variants. However, the demonstration of the role of Sigma-1R mutation in the context of homozygous cells with endogenous level of *SIGMAR1* expression has been never addressed so far.

Our work fills this gap, providing the first evidence of the cellular and molecular effects of the dHMN-linked Sigma-1R^E150K^ variant in human primary cell cultures derived from dHMN patients. Our findings clearly point to a pleiotropic role of Sigma-1R in the maintenance of cellular homeostasis, which involves the modulation of Ca^2+^ signalling, intracellular organelle contacts, mitochondrial metabolism and proteostasis and, importantly, recognize it as a potential targetable molecule for the development of novel therapeutic strategies to treat dHMN and related pathologies.

## Results

### Sigma-1R shows a significant mislocalization in cells from dHMN patients expressing the mutated Sigma-1R^E150K^ variant

**Fig. 1:**
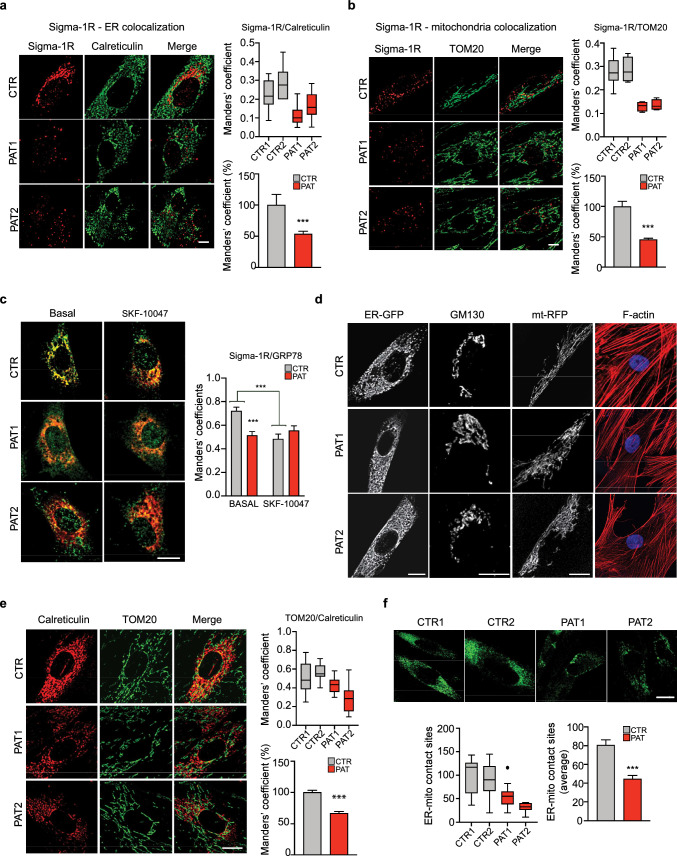
Altered localization of Sigma-1R protein in dHMN patient fibroblasts. **A**, **B** Representative confocal images of Sigma-1R (in red) and the ER-marker calreticulin (A) (in green), or the mitochondrial marker TOM20 (B) (in green) immunofluorescence staining in primary skin fibroblasts from healthy donor (CTR1) and Sigma-1R^E150K^ homozigous fibroblasts from dHMN patient (PAT1, PAT2). The graphs represent the colocalization of Sigma-1R to ER (A) or to mitochondria (B) expressed as Manders’ coefficient calculated for each individual (upper boxplot) or as average of the percentage relative to controls (bottom histogram). **C** Representative confocal images of Sigma-1R (in red) and GRP78 (in green) immunofluorescence staining in control (CTR) and dHMN patient (PAT1, PAT2) fibroblasts treated with vehicle (Basal) or with the Sigma-1R agonist SKF-10047 (10 μM) for 1 h. The graph represents the average Manders’ coefficients. **D** Representative confocal images of healthy donor (CTR) and dHMN patient (PAT1, PAT2) fibroblasts transfected with ER-targeted GFP (ER-GFP) or mitochondria-targeted RFP (mt-RFP) constructs, immunostained with Golgi specific antibody (GM130), or stained with fluorescent phalloidin to label F-actin (red). Hoechst is used to mark nuclei. **E** Representative immunostaining of TOM20 (in green) and calreticulin (in red) of cells as in A, B. The quantification of Manders’ coefficient is reported as in A, B. **F** Representative immunostaining of healthy donor (CTR1, CTR2) and dHMN patient (PAT1, PAT2) fibroblasts transfected with the ER-mitochondria-SPLICS probe. The graphs represent the quantification of SPLICS signal (puncta/cell) for each individual (left box plot) and as average percentage relative to control (right histogram). Data information: data of the box plot graphs reported in A–B and E–F are from at least ten confocal images and three independent experiments. Student’s t-test was used to assess statistical significance. Data are reported as mean ± SEM. *p < 0.05, ***p < 0.001. Scale bars = 10 μm

In the recent years, *SIGMAR1* has been found mutated in various familial cases associated with hereditary motor neuropathy and ALS forms [16–18, 22, 28–35, 38–40]. The available studies investigating the different Sigma-1R mutations revealed the constant presence of alteration in the localization of the mutant Sigma-1R proteins [30, 31, 34]. We have already shown that the overexpression of the dHMN-linked Sigma-1R^E150K^ mutation induces a significant mislocalization of the overexpressed protein [30]. In order to assess whether the distribution of endogenous Sigma-1R^E150K^ protein is affected in homozygous patient cells, we performed immunofluorescence analysis on skin fibroblasts from two patients bearing the homozygous c.448G > A (p.E150K) *SIGMAR1* mutation originally described in [30] and from healthy individuals as controls. Sigma-1R shows a significant colocalization with the ER marker calreticulin in control cells, as revealed by the Manders’ coefficient quantification (0.24 for Sigma-1R-calreticulin and 0.042 for calreticulin-Sigma-1R, see Fig. 1A and supplementary Fig. S1 A). Interestingly, Sigma-1R displays a marked reduction of colocalization with ER in patient-derived cells compared to controls (46.3% and 46,8% lower colocalization index for Sigma-1R-calreticulin and calreticulin-Sigma-1R, respectively, see Fig. 1A and supplementary Fig. S1 A), confirming the mislocalization of the mutant Sigma-1R^E150K^ protein in patient fibroblasts. Sigma-1R is described to associate with the intracellular microdomains at the contacts between ER and mitochondria called mitochondria-associated membranes (MAMs) in many neuronal and non-neuronal cell lines [1, 30]. We demonstrate here that Sigma-1R is found at the MAM also in primary human skin cells as revealed by the colocalization of endogenous Sigma-1R with the outer mitochondrial membrane (OMM) integral protein TOM20 in control fibroblasts (Manders’ coefficient = 0.26 for Sigma-1R-TOM20 and 0.11 for TOM20-Sigma-1R, see Fig. 1B and supplementary Fig. S1 B). Differently, fibroblasts from dHMN patients display a much lower Sigma-1R and TOM20 colocalization (Manders’ coefficient = 0.13 for Sigma-1R-TOM20 and 0.037 for TOM20-Sigma-1R, corresponding to a 54.4% and 64.6% reduction of Sigma-1R-TOM20 and TOM20-Sigma-1R colocalization compared to controls, respectively (see Fig. 1B and supplementary Fig. S1 B). This indicates that the endogenous Sigma-1R^E150K^ protein is mislocalized out of the MAMs, similarly to what previously observed with the overexpressed protein [30]. Sigma-1R has been shown to directly interact with the ER chaperone 78-kDa glucose-regulated protein (Grp78, also known as Bip) in basal conditions and this interaction is reversible and promptly released upon ER stress and Sigma-1R agonist addition [1]. We therefore assessed the association of Sigma-1R with its Grp78 partner by performing a double immunofluorescence staining with Sigma-1R and Grp78 antibodies in dHMN patients and control fibroblasts, before and after the addition of the Sigma-1R agonist SKF-10047 [41]. As shown in Fig. 1C, the degree of Sigma-1R-Grp78 association is high in untreated control cells, as revealed by the colocalization index of the two proteins (Manders’ coefficient = 0.72 for Sigma-1R-Grp78) and is significantly reduced upon Sigma-1R agonist stimulation (33,3% reduction), as expected (Fig. 1C). Differently, in dHMN patient cells, Sigma-1R and Grp78 interaction is relatively low already in basal conditions (the colocalization index is 29% lower than controls), and the effect of Sigma-1R agonist is nearly absent (Fig. 1C). Once again, these results point to a significant alteration of the mutant Sigma-1R^E150K^ protein localization and activity. Nevertheless, the overall ER morphology seems not affected by the expression of Sigma-1R^E150K^, as revealed by the distribution of the endogenous calreticulin protein and of an ectopically expressed GFP that was genetically targeted to the ER. Indeed, the signal of the two ER markers is comparable in dHMN patient and control fibroblasts (Fig. 1A, D). Similarly, the Golgi and mitochondria markers (GM130 and TOM20, respectively, Fig. 1B, E, D), as well as the overexpressed mitochondria-targeted red fluorescence protein (mtRFP, Fig. 1D), have a superimposable distribution in control and patient cells. This indicates that the presence, size and morphology of the major intracellular compartments are overall preserved in cells expressing the Sigma-1R^E150K^ protein. In addition, the cytoskeletal actin network is also unaffected in patient fibroblasts (Fig. 1D), ruling out the possible involvement of the Sigma-1R^E150K^ mutation in the regulation of cytoskeleton dynamics by its interaction with ankyrin, as suggested [42].

### Homozygous Sigma-1R^E150K^ fibroblasts from dHMN patients have reduced ER-mitochondria contacts

We have previously shown that the overexpression of dHMN-linked Sigma-1R mutant protein severely disturbs the distribution of MAM in neuroblastoma cells [30]. We then wondered whether the limited localization of Sigma-1R^E150K^ at ER-mitochondria contact sites in patient homozygous cells might also affect the extent of ER-mitochondria interaction. We assessed the number and distribution of MAM in primary cells from control individuals and dHMN patients using two different approaches. Firstly, we analysed the colocalization of mitochondria and ER organelles by Manders’ coefficient quantification in immunofluorescence experiments with anti-TOM20 and anti-calreticulin antibodies (Fig. 1E and supplementary Fig. S1 C). Secondly, we transfected cells with the recently described split-GFP-based SPLICS probe [43] and quantified the mitochondria-ER contacts as the number of GFP positive dots (Fig. 1F). In both cases, our results indicate a marked decrease of mitochondria-ER contact sites in homozygous Sigma-1R^E150K^ fibroblasts, with a 33% reduction of the TOM20-calreticulin, 17.3% reduction of the calreticulin-TOM20 Manders’ coefficients, and 55% reduction of the SPLICS probe signal, compared to controls. These findings support the notion that Sigma-1R mutation impinges on the establishment and/or maintenance of the close contact between the two organelles in patient cells.

### The reduced expression of Sigma-1R^E150K^ in patient cells is due to its enhanced protein degradation

**Fig. 2:**
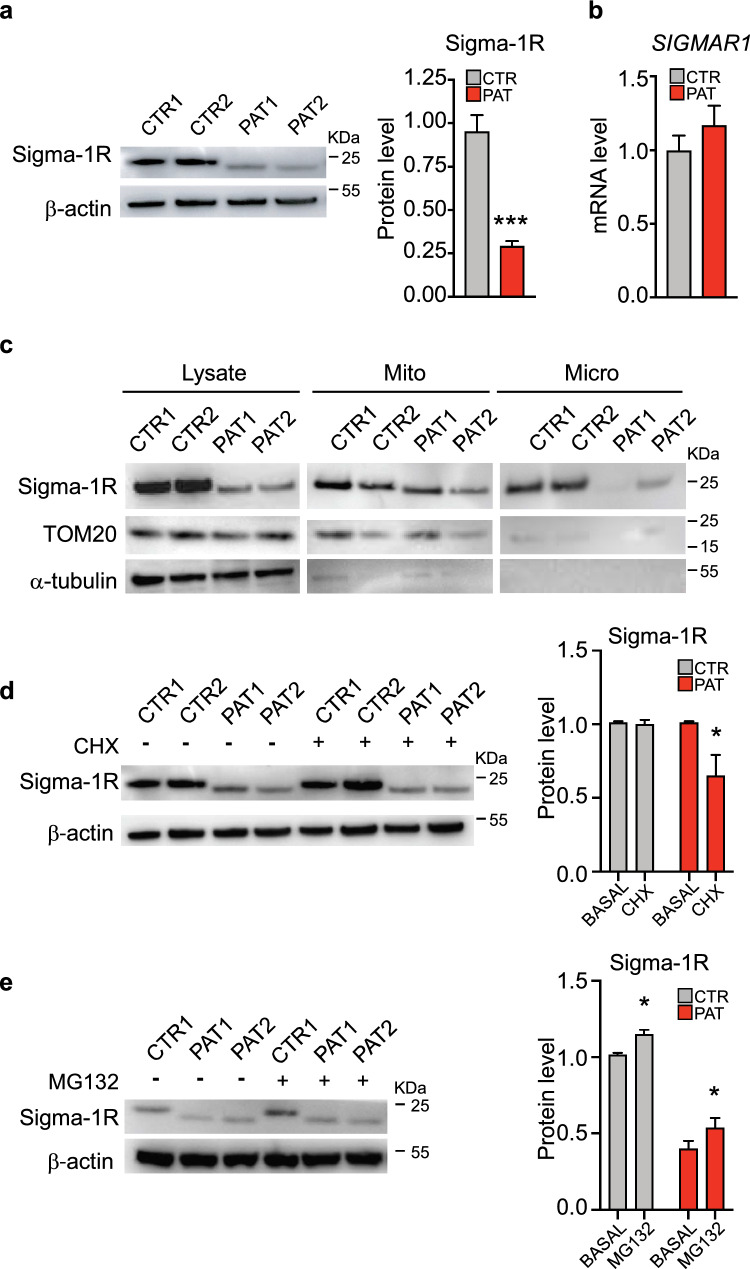
An increased turnover of Sigma-1R^E150K^ protein is responsible of its reduced level in dHMN patient fibroblasts. **A** Representative immunoblotting and relative quantification of Sigma-1R protein level in fibroblast lysates from healthy individuals (CTR1, CTR2) and dHMN patients (PAT1, PAT2). β-actin is used as loading control. **B** The histogram represents the real-time PCR quantification of *SIGMAR1* mRNA level in healthy individual (CTR) and dHMN patient (PAT) fibroblasts, normalized to *Actb*. **C** Representative immunoblotting of total lysate (Lysate), mitochondrial (Mito) and microsomal (Micro) protein fractions from cells as in A. TOM20 is used as marker of mitochondria, α-tubulin is used as loading control. **D** Representative immunoblotting and relative quantification of Sigma-1R protein level from cells as in A treated with vehicle (BASAL) or 5 μM cycloheximide (CHX) for 24 h. β-actin is used as loading control. **E** Representative immunoblotting and relative quantification of Sigma-1R protein level in lysates from control (CTR1) and patient (PAT1, PAT2) fibroblasts treated with vehicle (BASAL) or 1 μM MG-132 for 4 h. β-actin is used as loading control. Data information: data reported in A are from fifteen independent experiments. Data in B-E are from three independent experiments. Data are presented as mean ± SEM. *p < 0.05

Our immunofluorescence analysis, besides evidencing the mislocalization of Sigma-1R^E150K^ protein, also highlighted a remarkable reduction of the overall expression of the mutated protein compared to the wild-type (wt) Sigma-1R protein (Fig. 1A, B). In order to quantify the endogenous level of Sigma-1R, we performed Western blot analysis of fibroblast cell lysates. We observed a significant reduction of the Sigma-1R protein level in patient cells respect to controls (69,7% reduction, see Fig. 2A) while the level of *SIGMAR1* transcripts did not show significant difference between patient and control samples (Fig. 2B). These findings indicate that the downregulation of mutant Sigma-1R^E150K^ is likely due to a post-transcriptional mechanism, as also suggested for other Sigma-1R pathogenic mutations [31, 34]. Subsequently, we conducted a cellular fractionation analysis in order to determine the distribution of Sigma-1R proteins in the different cell compartments (cytoplasm, mitochondria and microsomal membranes, see Fig. 2C). The amount of Sigma-1R^E150K^ is reduced in all cellular fractions obtained from patient fibroblasts, with the strongest Sigma-1R downregulation occurring in the microsomal membranous fraction, which mainly consists of ER membranes [44] (Fig. 2C). Once more, these results highlight a significant mislocalization of the mutant protein from its canonical location at the ER membranes.

Two possible mechanisms may be responsible for the observed reduction of Sigma-1R protein expression in patient cells: (i) a defective synthesis or (ii) an enhanced degradation. In order to discriminate between these two possibilities, we inhibited cellular protein synthesis by cycloheximide (CHX, 5 µM) and monitored Sigma-1R protein degradation rate after 24 h. The quantification of Sigma-1R level by Western blotting in treated cells reveals that the treatment does not significantly reduce Sigma-1R amount in control fibroblasts (Fig. 2D), meaning that the degradation of the endogenous protein is relatively slow and not appreciable under these conditions. On the contrary, homozygous Sigma-1R^E150K^ fibroblasts display a significant decrease of Sigma-1R expression after CHX, pointing to a more rapid turnover of the mutant protein in patient cells respect to controls (Fig. 2D). This was also confirmed by overexpression experiments in a different cellular model, where HEK-293T cells were transfected with wt Sigma-1R and Sigma-1R^E150K^ coding plasmids. The overexpressed Sigma-1R^E150K^ showed a more rapid degradation after CHX treatment compared to the overexpressed wt protein also in HEK-293T cells (supplementary Fig. S2 A), similarly to what observed for the endogenous protein in primary fibroblasts. We then investigated whether the ubiquitin–proteasome system could be the possible degradative pathway responsible for the increased clearance of mutant Sigma-1R in patient cells. We then treated dHMN and control fibroblasts, as well as HEK-293T cells expressing wt Sigma-1R and Sigma-1R^E150K^, with the proteasome inhibitor MG-132 and evaluated the amount of Sigma-1R after 8 h. If the proteasome was involved in the degradation of mutant Sigma-1R, dHMN fibroblasts and HEK-293T cells overexpressing Sigma-1R^E150K^ should display an increased Sigma-1R level after MG-132 treatment. Our results demonstrated that endogenous Sigma-1R protein is not augmented in MG132-treated patient and control fibroblasts (Fig. 2E). Similarly, neither Sigma-1R^E150K^ nor wt Sigma-1R levels did change in overexpressing HEK-293 T cells after incubation with MG-132 (supplementary Fig. S2 B). This may indicate that a different degradative mechanism, other than proteasome, accounts for the reduced Sigma-1R^E150K^ expression in patient cells.

### The autophagy-lysosomal pathway is enhanced in dHMN patient cells expressing Sigma-1R^E150K^

**Fig. 3:**
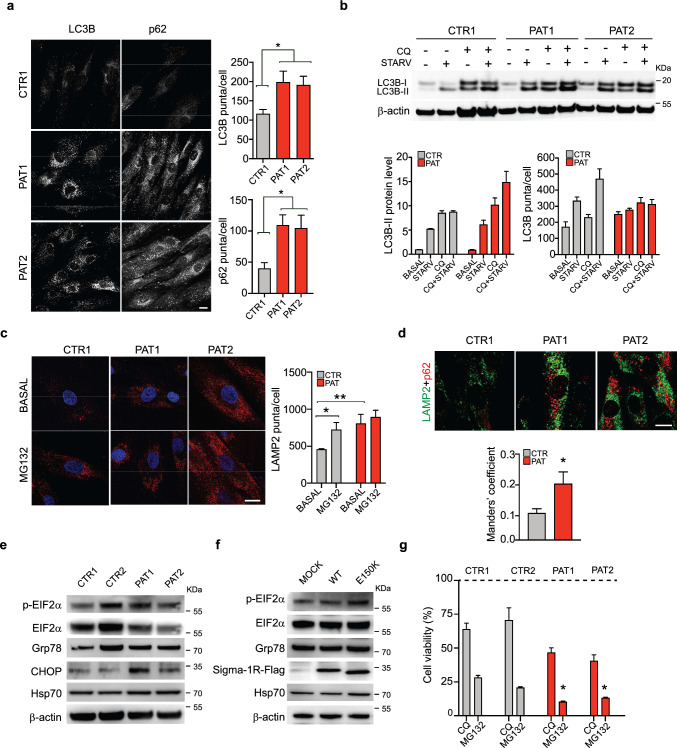
Homozygous Sigma-1R^E150K^ fibroblasts have enhanced authophagy-lysosomal and ER stress pathways. **A** Representative confocal images of LC3B and p62 immunostaining in control (CTR1) and patient (PAT1, PAT2) fibroblasts. The histograms report the average number of LC3B and p62 puncta per cell. **B** Representative immunoblotting of LC3B level in control (CTR1) and patient (PAT1, PAT2) fibroblasts in standard conditions or after 4 h of starvation in PBS (STARV), with or without chloroquine (CQ, 50 μM for 1 h). β-actin was used as loading control. The histograms report the quantification of LC3B-II immunoblot band intensity normalized for that of β-actin (left) and of the number of LC3B-positive autophagosomes per cell calculated from images in Supplementary Fig. S3. **C** Representative confocal images of LAMP2 immunostaining in control (CTR1) and patient (PAT1, PAT2) fibroblasts treated with or without the proteasome inhibitor MG-132 (1 μM, for 4 h). The histogram reports the average number of LAMP2 dots per cell. **D** Representative confocal images of LAMP2 (in green) and p62 (in red) immunostaining in control (CTR1) and patient (PAT1, PAT2) fibroblasts. The graph represents the colocalization of LAMP2 to p62 expressed as Manders’ coefficient. **E**, **F** Representative immunoblotting of the indicated ER-stress markers and of the Flag-tagged Sigma-1R protein in lysates from control (CTR1, CTR2) and patient (PAT1, PAT2) fibroblasts (E) or from HEK293T cells transfected with empty (MOCK), WT Sigma-1R-Flag or Sigma-1R^E150K^-Flag expressing constructs for 24 h (F). β-actin was used as loading control. **G** Cell viability measured by MTS assay in healthy individual (CTR1, CTR2) and patient (PAT1, PAT2) fibroblasts treated with or without chloroquine (CQ, 50 μM for 6 h) or MG-132 (2 μM for 24 h) expressed as percentage relative to non-treated cells. Data information: The results are representative of at least two (panels B, C and D), three (panels A and G), five (panels E and F) independent experiments. Data of the graphs reported in A, C and D are from at least 10 confocal images. Data are presented as mean ± SEM. *p < 0.05, **p < 0.01. Scale bars = 10 μm

We have previously demonstrated that the two dHMN-associated *SIGMAR1* mutations (c.412G > C p.E138Q and c.448G > A p.E150K) caused alteration in autophagy flux when overexpressed in SH-SY5Y cells [30]. We wondered whether homozygous fibroblasts expressing endogenous levels of the Sigma-1R^E150K^ variant display a similar phenotype. The immunostaining of control and patient fibroblasts with antibodies for the two classical autophagosome markers, LC3 and p62, showed a higher signal in dHMN patient cells compared to controls, i.e. 1.4-fold and 2-fold more LC3- and p62-positive staining, respectively (Fig. 3 A). This confirms that Sigma-1R^E150K^ expression enhances autophagosome formation (Fig. 3 A). In addition, we showed that the increased autophagosome number in patient fibroblasts is not due to a block of the autophagic flux but rather to its enhancement. In fact, both starvation and chloroquine, which are canonical inducers of autophagosome formation, stimulate the accumulation of LC3B and p62 proteins and the formation of LC3 positive autophagosome vesicles in both cell types. As expected, the combination of the two treatments has a stronger effect compared to either condition alone, and this additive effect is appreciable in both control and patient fibroblasts (Fig. 3B and supplementary Fig. S3 A, B). Subsequently, we wondered whether the increased autophagy in dHMN cells was accompanied by an enhanced lysosomal activity. We analysed lysosome distribution and number by staining cells with the lysosomal marker LAMP2. As shown in Fig. 3C, patient fibroblasts evidence a significant increased LAMP2 positivity compared to controls. In addition, control cells show a remarkable upregulation of LAMP2-positive vesicles after addition of the proteasome inhibitor MG-132, as expected since it stimulates the lysosomal pathway [45]. Differently, the LAMP2 signal is not augmented in MG-132 treated fibroblasts homozygous for Sigma-1R^E150K^ (Fig. 3C). This suggests that the lysosomal system is already at its highest activity in patient cells, thus preventing any additional stimulation/increase. Finally, the co-staining of control and Sigma-1R^E150K^ expressing fibroblasts with p62 and LAMP2 clearly showed an enhanced colocalization of the two markers in cells expressing mutant Sigma-1R respect to controls, as indicated by the increased Manders’ coefficient (1.8-fold, Fig. 3D). We conclude that patient fibroblasts have more autophagosomes and lysosomes that are in close proximity and eventually fuse to each other. Thus, in conclusion, dHMN cells have a significantly upregulated autophagy-lysosomal pathway compared to controls.

### Homozygous Sigma-1R^E150K^ fibroblasts have increased levels of CHOP and phosphorylated-eIF2α ER stress markers

Mutations in Sigma-1R protein found in neurodegenerative disease cases, as well as Sigma-1R downregulation, are normally associated with the induction of the cellular ER stress response [1, 31, 34, 46–48]. Three main intracellular effectors and the downstream signalling pathways are involved in the activation of the ER stress response: PERK, IRE1 and ATF6 [49]. We investigated all these three branches by monitoring: (i) eIF2α phosphorylation and ATF4 expression, downstream of PERK; (ii) the spliced variant of XBP, downstream of IRE1 and (iii) the expression of selected genes downstream of ATF6, such as *EDEM* and *XBP*, in patient and control cells. We did not find upregulation of total *XBP* (*XBPtot*), spliced *XBP* (*sXBP*), *ATF4*, and *EDEM* mRNA levels in fibroblasts expressing Sigma-1R^E150K^. On the contrary, *EDEM* transcripts appear to be reduced compared to controls (supplementary Fig. S3 C). In addition, two ER chaperones, the 70-kDa heat shock protein (Hsp70) and Grp78, which are normally induced upon ER stress, are not significantly increased in patient cells compared to controls (Fig. 3E and supplementary Fig. S3 D). Despite that, eIF2α, which is the primary target of PERK kinase, is remarkably more phosphorylated in patient cells expressing Sigma-1R^E150K^ compared to controls. In addition, the eIF2α protein level is reduced in Sigma-1R^E150K^ fibroblasts thus strongly enhancing the ratio of phospho-eIF2α over total eIF2α in these cells (Fig. 3E and supplementary Fig. S3 D). Interestingly, also the C/EBP homologous protein (CHOP), which is the terminal effector of the ER stress-induced transcriptional program, is significantly upregulated in patient fibroblasts compared to controls (Fig. 3E and supplementary Fig. S3 D). The ectopic expression of Sigma-1R constructs in HEK-293T cell line confirmed that the phosphorylation of eIF2α and the induction of CHOP are specifically increased by the expression of mutant Sigma-1R^E150K^ and are not a consequence of patient cell adaptation to the culture conditions (Fig. 3F and supplementary Fig. S3 E). Interestingly, we found that the level of Hsp70 protein is higher in HEK-293T cells overexpressing Sigma-1R^E150K^ respect to those expressing wt Sigma-1R, as we already reported in neuronal cell lines [30], while there is not difference between homozygous Sigma-1R^E150K^ and control fibroblasts. In conclusion, patient cells homozygous for the Sigma-1R^E150K^ mutation display a strong upregulation of the autophagy-lysosomal degradative system together with the induction of specific ER stress response markers. Overall, these data suggest that the presence of mutated Sigma-1R protein causes a general derangement of protein homeostasis and protein clearance in patient cells, which may, at least partially, explain the abnormal Sigma-1R distribution observed in the homozygous Sigma-1R^E150K^ fibroblasts.

### Homozygous Sigma-1R^E150K^ fibroblasts show increased susceptibility to proteostatic insults

We previously showed that overexpression of the dHMN-linked Sigma-1R mutations (E150K and E138Q) in neuronal cell lines leads to abnormal Sigma-1R protein distribution, enhanced autophagosome formation and increased susceptibility to cell death [30]. We wondered whether the endogenous expression of Sigma-1R^E150K^ in homozygous patient fibroblasts also impacts cell survival.

We then challenged homozygous Sigma-1R^E150K^ and control fibroblasts with different cell death inducing stimuli and analysed cell viability at 24 h. Surprisingly, both in basal condition and after addition of classical apoptotic inducers (staurosporine, starvation), ER stressors (thapsigargin, tunicamycin), and oxidative stress induction (H_2_O_2_, antimycin and rotenone), we did not observe differences in cell viability between control and patient cells (not shown). However, patient cells display a significantly reduced viability respect to controls in the presence of treatments impinging on cell proteostasis, such as chloroquine and MG-132 (Fig. 3G). This points to a relatively mild effect of the endogenous homozygous expression of Sigma-1R^E150K^ on cell viability in resting conditions and, importantly, to a specific susceptibility of homozygous patient cells to insults that affect protein quality control and protein clearance.

### Global calcium homeostasis is perturbed in dHMN patient cells

**Fig. 4:**
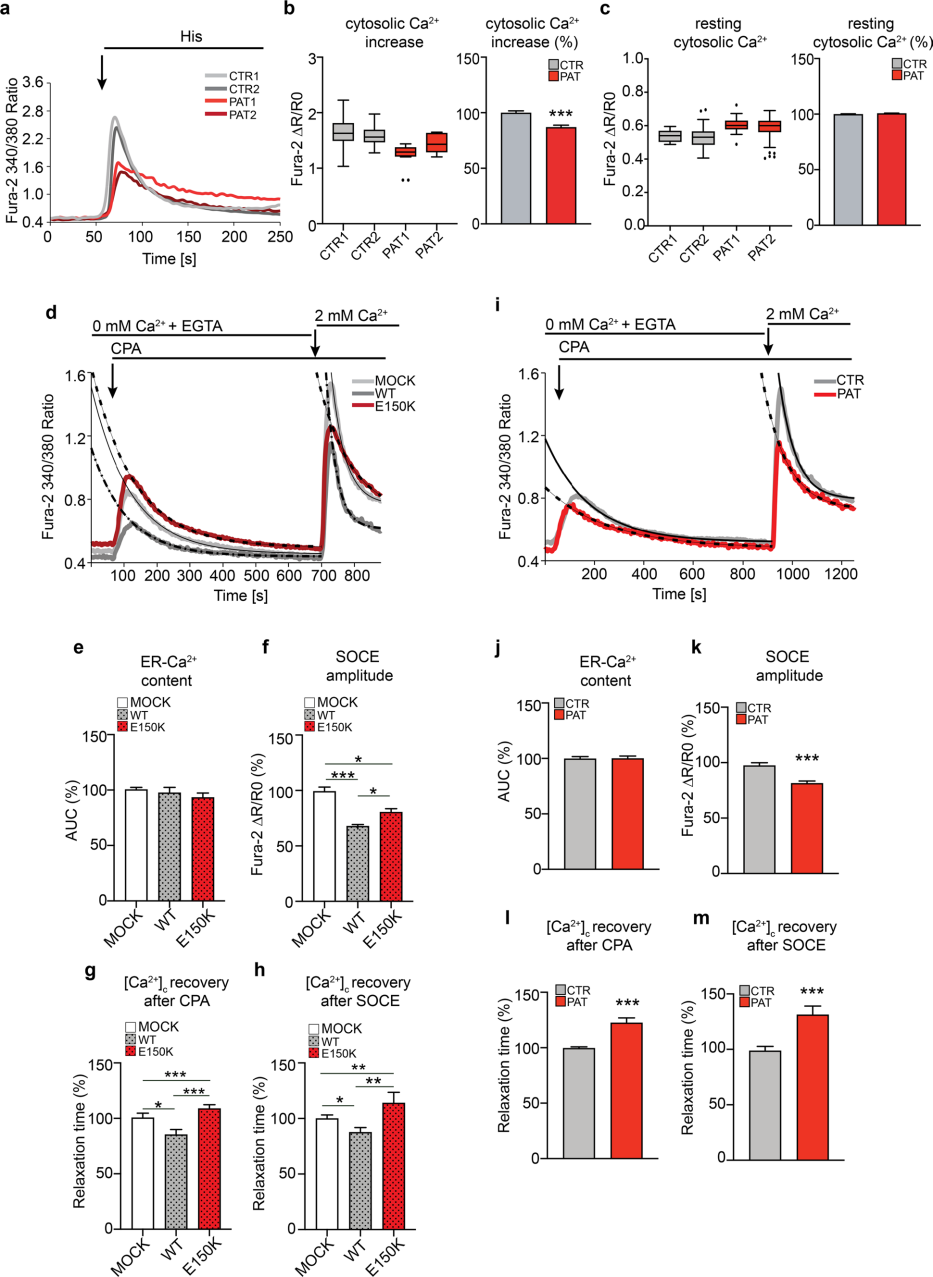
Derangement of global intracellular Ca^2+^ handling in Sigma-1R^E150K^ expressing fibroblasts. **A** Representative traces of cytosolic Ca^2+^ measurements with Fura-2AM in control (CTR) and dHMN patient (PAT) fibroblasts challenged with the ER-Ca^2+^-release agonist histamine (His 100 μM). **B**, **C** The graphs represent the quantification of the cytosolic Ca^2+^ levels for each individual expressed as Fura-2-AM ΔR/R0 values (box plots) or as average percentage of the ΔR/R0 relative to controls (histograms) after histamine stimulation (B) or in resting condition (C). **D** Representative traces of cytosolic Ca^2+^ measurements with Fura-2-AM in fibroblasts from healthy donor in Ca^2+^-free conditions, transfected with empty vector (MOCK), WT or Sigma-1R^E150K^ constructs for 24 h. ER-Ca^2+^release was induced by CPA (5 μM) and SOCE triggered by addition of 2 mM Ca^2+^. Black lines represent the exponential fit of the cytosolic Ca^2+^ recovery after CPA or SOCE, respectively. **E**, **H** Histograms report the average of ER-Ca^2+^ content calculated as the area under the curve (AUC) after CPA addition (E), the SOCE amplitude after 2 mM Ca^2+^ addition (F), the rate of cytosolic Ca^2+^ ([Ca^2+^]_c_) recovery after CPA (G) and after SOCE (H) from experiments as in D and expressed as percentage relative to mock. See supplementary Fig. S4 A for the details of parameter calculations. **I** Representative traces of cytosolic Ca^2+^ measurements with Fura-2A-M in control and patient fibroblasts in Ca^2+^ free condition performed as in D. **J**, **M** Histograms report the average of ER-Ca^2+^ content calculated as the area under the curve (AUC) after CPA addition (J), the SOCE amplitude after 2 mM Ca^2+^ addition (K), the rate of cytosolic Ca^2+^ ([Ca^2+^]_c_) recovery after CPA (L) and after SOCE (M) from experiments as in (I) in control (CTR) and patient (PAT) fibroblasts. Data information: Data in B, C are from 11 independent experiments. Data in E–H are from three independent experiments. Data in J-M are from five independent experiments. See scheme in supplementary Fig. S4 A for the complete description of parameters used in E–H and J-M. Data are shown as the means ± SEM. Student’s t-test was used for statistical significance assessment. *P < 0.05 **P < 0.001, ***P < 0.0001. Black circles represent the outliers

Considering the marked impact of homozygous Sigma-1R^E150K^ expression on mitochondria-ER contacts, and previous data from our and other groups on the effect of Sigma-1R mutation or downregulation on cellular Ca^2+^ homeostasis [7, 30, 31], we analysed in details the intracellular Ca^2+^ dynamics of dHMN patient fibroblasts. We measured the Ca^2+^ concentration ([Ca^2+^]_i_) by Fura-2-AM dye in the cytosol of patient and control cells both in resting condition and after IP3-generating agonist (histamine) stimulation. Interestingly, the [Ca^2+^]_i_ transient after histamine addition was significantly lower in Sigma-1R^E150K^ patient fibroblasts (14% reduction) compared to controls (Fig. 4A, B), in line with what previously observed in neuronal cells ectopically expressing Sigma-1R^E150K^ [30]. However, the resting [Ca^2+^]_i_ did not differ among samples (Fig. 4A, C). We reasoned that the reduced IP3-evoked [Ca^2+^]_i_ transient in patient fibroblasts may be due to a diminished activity of the IP_3_R channels, which are the main ER Ca^2+^ release channels in most cell lines. In order to test this hypothesis, we monitored the expression level of the IP_3_R3, which is highly expressed in cultured cells including fibroblasts [50] and is the direct client of Sigma-1R chaperone activity at the ER membrane [2, 42]. Interestingly, our Western blot data showed a marked reduction of the IP_3_R3 receptor in patient cells compared to controls (supplementary Fig. S4 B). In addition, in order to dissect all the possible contributions to the impaired [Ca^2+^]_i_ elevation in cells expressing mutated Sigma-1R, we quantified the ER Ca^2+^ content and the rate of Ca^2+^ influx through the plasma membrane after depletion of the Ca^2+^ stores in order to evaluate the so called store operated Ca^2+^ entry (SOCE). Briefly, we firstly monitored the [Ca^2+^]_i_ in control fibroblasts overexpressing either wt Sigma-1R or Sigma-1R^E150K^ constructs in Ca^2+^-free conditions. Then, we emptied the ER-Ca^2+^ store adding the SERCA inhibitor cyclopiazonic acid (CPA), and finally we challenged cells with 2 mM Ca^2+^ to induce Ca^2+^ influx through plasma membrane. As shown in Fig. 4D, E and supplementary Fig. S4 C, we did not observe significant difference in the overall ER Ca^2+^ content ([Ca^2+^]_ER_) between Sigma-1R^E150K^ and wt Sigma-1R transfected fibroblasts (the measure of [Ca^2+^]_ER_ was obtained by the area under the curve after CPA addition, all the methods and parameters are detailed in supplementary Fig. S4 A). Nevertheless, wt Sigma-1R overexpression induces a strong decrease of SOCE peak (33.2% inhibition compared to empty vector) (Fig. 4D, F and supplementary Fig. S4 D) and SOCE rate (time to peak, not shown), in line with previously reported data [7]. Of note, Sigma-1R^E150K^ expression leads to only a partial inhibition of SOCE (20.1% inhibition compared to empty vector) supporting the notion that the E150K substitution behaves as a loss-of-function mutation and impairs Sigma-1R regulation of Ca^2+^ influx (Fig. 4 D, F and supplementary Fig. S4 D).

Interestingly, the rate of [Ca^2+^]_i_ recovery after both CPA and Ca^2+^ addition is significantly slower in control fibroblasts transfected with Sigma-1R^E150K^ compared to cells transfected with wt Sigma-1R, as indicated by the higher τ value (22.8% and 27.4% increase after CPA and SOCE, respectively, where τ = time to reach 1/*e*, or ~ 37%, of the peak [Ca^2+^]_i_ value, as calculated by the exponential fit of the [Ca^2+^]_i_ decay, see Materials and Methods for details) (Fig. 4D, G, H and supplementary Fig. S4 A, E, F). Similar results were obtained from the analysis of the [Ca^2+^]_ER_ and SOCE in homozygous Sigma-1R^E150K^ patient fibroblasts. Indeed, the ER Ca^2+^ release upon SERCA inhibition is comparable between patient and control cells (Fig. 4I, J and supplementary Fig. S4 G), while the [Ca^2+^]_i_ clearance after both CPA and extracellular Ca^2+^ addition is delayed in patient cells, as indicated by the increased τ value, by 22.4% after CPA and by 31,1% after Ca^2+^ addition, compared to controls (Fig. 4I, L, M and supplementary Fig. S4 I, J).

Surprisingly, the effect of Sigma-1R^E150K^ expression on SOCE amplitude is different between homozygous and overexpressing fibroblasts. Indeed, SOCE peak (Fig. 4I, K and supplementary Fig. S4 H) and SOCE rate (not shown) are significantly reduced in patient fibroblasts compared to controls (19.7% peak amplitude reduction). Rationally, one would expect that a less functional Sigma-1R protein (such as the Sigma-1R^E150K^ mutant) would relieve the Sigma-1R-mediated STIM1 inhibition and thus enhance SOCE. We might explain these unpredicted results by a perturbation of Sigma-1R activity on the STIM1/ORAI1 pair due to the E150K mutation. This may affect STIM1/ORAI1 protein expression, localization or complex formation in a sort of gain-of-toxic-function mechanism; however, more experiments are needed to clarify the issue. In addition, we hypothesize that this toxic function of Sigma-1R^E150K^ on SOCE regulation may contrast and overcome the effect of the reduced Sigma-1R protein level in patient fibroblasts, which would per se enhance Ca^2+^ influx. Despite the molecular identification of this regulatory circuit remains unclear and needs to be further investigated, the use and the analysis of patient-derived cells were instrumental to reveal it, since it could not be otherwise appreciated in overexpression systems, where the endogenous protein is still present and may mask the effect of the mutation.

### Sigma-1R^E150K^ expression impairs mitochondrial Ca^2+^ uptake and aerobic metabolism in dHMN patient cells

**Fig. 5:**
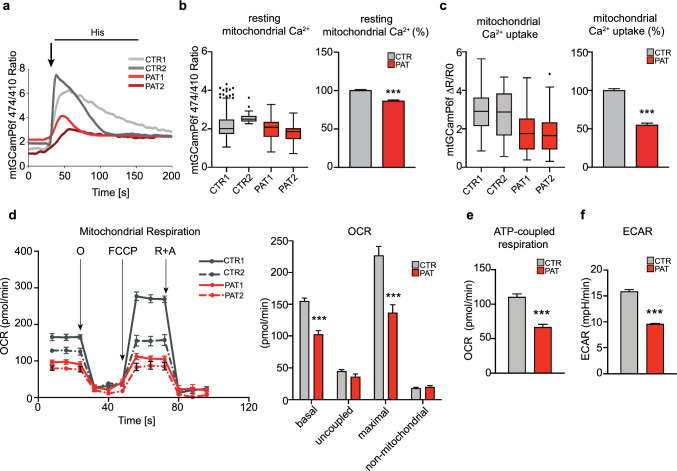
Impairment of mitochondrial Ca^2+^ signaling and metabolism in dHMN fibroblasts. **A** Representative traces of mitochondrial Ca^2+^ measurements obtained with the 4mt-GCaMP6f indicator in control (CTR) and dHMN patient (PAT) fibroblasts challenged with histamine (His 100 µM). **B**, **C** The graphs represent the quantification of the mitochondrial Ca^2+^ level measured with the 4mt-GCaMP6f probe for each individual (box plots) or as average percentage relative to controls (histograms) in resting condition (expressed as 474/410 nm ratio, B) and after histamine stimulation (expressed as ΔR/R0, C). **D** Representative traces and relative quantification of the oxygen consumption rate (OCR) in healthy donor (CTR1, CTR2) and dHMN patient (PAT1, PAT2) fibroblasts. Oligomycin (O) is used to determine ATP synthesis-coupled respiration; the proton uncoupler carbonyl cyanide-4-(trifluoromethoxy)phenylhydrazone (FCCP) is used to determine the maximal and the spare respiratory capacity, and the respiratory complex I and III inhibitors rotenone and antimycin A (R + A) are used to determine non-mitochondrial respiration. **E** Measurement of the ATP-coupled respiration extracted from the OCR data presented in D. **F** Quantification of the extracellular acidification rate (ECAR). Data information: Data in B, C are from 5 independent experiments. Seahorse data are from three independent experiments. Data are reported as mean ± SEM values. ***P < 0.001. Black circles represent the outliers

Considering the significant derangement of the intracellular Ca^2+^ dynamics observed in patient cells and the fact that mutant Sigma-1R^E150K^ disrupts the ER-mitochondria contacts responsible for the Ca^2+^ transfer from the ER store to the mitochondria, we hypothesized that the homozygous expression of Sigma-1R^E150K^ would impinge on the mitochondrial Ca^2+^ homeostasis of dHMN fibroblasts. Thus, we monitored the Ca^2+^ concentration in mitochondria of patient and control cells, in both resting conditions and after an IP_3_-mobilizing agonist stimulation (histamine) using a mitochondria-targeted version of the Ca^2+^-sensitive GCaMP6f probe. Our data clearly demonstrated that both basal mitochondrial Ca^2+^ concentration (Fig. 5A, B) and histamine-induced mitochondrial Ca^2+^ uptake (Fig. 5A, C) are significantly reduced in dHMN patient fibroblasts compared to controls (13.3% and 45.8% reduction, respectively), in line with what we reported in cell lines overexpressing Sigma-1R^E150K^ [30]. We excluded that this may be due to the downregulation of the mitochondrial Ca^2+^ uniporter (MCU) components, since the expression of its major constituents MCU, Micu1 and Micu2 [51] shows no difference between patient and control cells at both the mRNA and protein level (supplementary Fig. S5 A, B). Subsequently, we investigated whether the blunted mitochondrial Ca^2+^ response of dHMN fibroblast mitochondria may influence the oxidative metabolism of the patient cells. As expected, they have lower respiratory capacity with a significant decrease of both basal and maximal oxygen consumption rate compared to controls (Fig. 5D), which correlates with a marked reduction of ATP-synthesis coupled respiration (Fig. 5E). The analysis of respiratory complex expression did not reveal major differences in patient versus control samples, except for complex I, which is significantly downregulated in dHMN fibroblasts (supplementary Fig. S5 C). In parallel, the rate of glycolysis, measured as the extent of the extracellular milieu acidification (ECAR), showed a remarkable downregulation of global energy metabolism of patient cells (Fig. 5F). Nevertheless, despite the defect in overall mitochondrial respiratory and metabolic activity, homozygous Sigma-1R^E150K^ patient cells do not show a significant increase of ROS production (supplementary Fig. S5 D).

Finally, we evaluated the intracellular membrane and organelle ultrastructure of patient and control cells through transmission electron microscopy (TEM). This analysis confirmed the reduction of mitochondria-ER close contacts (supplementary Fig. S5 E, left) and revealed the presence of alterations in the mitochondria cristae morphology (represented by the enlargement and disorganization of the cristae array, see supplementary Fig. S5 E, left) while rough ER and Golgi apparatuses do not show remarkable differences among genotypes. Moreover, we observed a slight but reproducible swelling of the nuclear envelope in dHMN patient samples that was absent in control preparations. Nuclear envelope enlargement was also reported in cells expressing the Sigma-1R^E102Q^ mutation associated to a juvenile ALS form (jALS) [31], which showed a far more severe alteration than what observed in our dHMN patient cells.

Concluding, the characterization of dHMN patient primary cells harbouring the homozygous Sigma-1R^E150K^ mutation revealed a significant mislocalization of the mutant Sigma-1R protein out of MAM and ER membranes. Despite that, no major morphological alterations of the intracellular compartments are evident. Finally, our results clearly pointed to an overall derangement of the inter-organelle communication and, in particular, a significant reduction of the ER-mitochondria interaction. In our opinion, this heavily contributes to the global impairment of intracellular and mitochondrial Ca^2+^ signalling and of mitochondria energy metabolism observed in Sigma-1R^E150K^ fibroblasts, thus representing a likely explanation for motor neuron dysfunction and degeneration in dHMN patients.

## Discussion

Sigma-1R is a ubiquitously expressed integral ER membrane protein with chaperone domain, endowed with pleiotropic cellular functions, ranging from the support of protein folding and stability, to the regulation of ion channel activity [2, 5, 52–55], to the maintenance of the nuclear lamina structure and chromatin remodelling [21, 38]. Despite the significant advances in the comprehension of Sigma-1R functions, important questions on the biology of this protein, in particular in the context of neurodegenerative pathologies such as dHMN, are still to be answered.

In order to clarify these issues, we performed an extensive characterization of a cellular model of dHMN, which consists of skin primary cells from patients harbouring the homozygous Sigma-1R^E150K^ mutation. This Sigma-1R variant has been demonstrated to cause dHMN in Italian families [30]. Our approach focused on the biochemical, morphological, and metabolic analysis of homozygous Sigma-1R^E150K^ patient fibroblasts and represents the first comprehensive investigation of the effects of endogenous levels of the mutant Sigma-1R protein on the intracellular signalling and mitochondrial metabolism in a human dHMN cell model.

Our findings demonstrated that the Sigma-1R^E150K^ variant expressed in dHMN patient cells displays a significant mislocalization from the typical presence in ER membranes and MAMs of the endogenous protein. This is in line with published reports on other HMN-associated Sigma-1R mutations [28, 30, 31, 34]. However, the Sigma-1R^E150K^ mislocalization is not associated to major alterations of the intracellular compartments’ structure, since the ER and Golgi networks display a morphology and distribution that are similar in homozygous dHMN patient and control cells. Nevertheless, the nuclear envelope of dHMN patient cells shows a subtle but reproducible expansion compared to cells from healthy individuals. A nuclear envelope enlargement, to a much higher degree, was also reported in homozygous lymphoblastoid cells expressing the jALS-linked Sigma-1R^E102Q^ variant [31]. To note, the expression of Sigma-1R^E102Q^ mutation induces a remarkable swelling of the ER cisternae and Golgi vesicles [31], which was not observed in Sigma-1R^E150K^ homozygous fibroblasts. The relatively less severe phenotype of Sigma-1R^E150K^ compared to Sigma-1R^E102Q^ homozygous cells may reflect the milder clinical phenotype of the Italian dHMN compared to the jALS patients [17, 30] and suggests a Sigma-1R site-specific mutation effect on both the molecular and clinical phenotypes.

Moreover, the analysis of the intracellular organelle distribution in dHMN fibroblasts, revealed a dramatic impairment of MAM structures in patient fibroblasts, confirming what previously found in cell lines overexpressing Sigma-1R^E150K^ [30]. This highlights a fundamental role of Sigma-1R in the modulation of ER-mitochondria communication, thus impacting on the crucial functions of MAMs, which include Ca^2+^ signalling, lipid metabolism and autophagy regulation [56, 57]. Indeed, MAMs act as pleiotropic hubs for the integration of different external and internal stimuli decoded as Ca^2+^ elevation. They are the site of ER-Ca^2+^ release and of mitochondria Ca^2+^ uptake in response to cell stimulation thus behaving as key modulators of cellular Ca^2+^ signals. Moreover, Ca^2+^ entry into mitochondria stimulates the respiratory chain and promotes cell aerobic metabolism [58], thus MAM function is also involved in the modulation of cellular oxidative metabolism. Sigma-1R role in the modulation of Ca^2+^ signal was previously suggested by studies mainly based on *SIGMAR1* silencing [7, 59] or overexpression of mutant constructs [30, 31]. In this context, for the first time, our study investigated the consequences of specific Sigma-1R mutation on global cell homeostasis, subcellular Ca^2+^ dynamics and mitochondrial metabolism in the context of endogenous levels of the mutant protein, in primary cells obtained directly from dHMN patients.

We observed a dramatic reduction of Sigma-1R protein levels in patient cells expressing Sigma-1R^E150K^. The downregulation of Sigma-1R protein expression results to be a common feature of Sigma-1R pathological variants, as shown for different *SIGMAR1* gene mutations [28, 31, 34]. In addition, our results indicate that the downregulation of Sigma-1R^E150K^ occurs at the post-transcriptional level, in line to what observed for other Sigma-1R variants (c.151 + 1G > T splicing site and E102Q jALS variants [28, 31, 34]) and showed that the decreased Sigma-1R^E150K^ content in patient cells is due to an enhanced protein degradation rather than a reduced protein synthesis. Furthermore, we excluded that the enhanced degradation of Sigma-1R^E150K^ may be ascribed to an increased proteasome activity that instead was previously implicated in the degradation of other Sigma-1R variants (c.151 + 1G > T [28] and p.N167I [34]). Indeed, the proteasome inhibitor has no effect in restoring Sigma-1R^E150K^ level in dHMN patient fibroblasts nor in HEK-293T cells overexpressing the mutant protein. This suggests the existence of distinctive pathogenic mechanisms for the different Sigma-1R mutant proteins, which may contribute to the differences in the cellular phenotype and clinical outcomes of the affected individuals.

The increased number of autophagosomes and lysosomes as well as their enhanced colocalization in dHMN patient fibroblasts suggested that the autophagy-lysosomal system is likely involved in the degradation of Sigma-1R^E150K^ in this context. Whether the observed enhanced autophagy-lysosomal activity is associated with or caused by an impaired/reduced proteasome function in dHMN cells remains to be elucidated.

Interestingly, fibroblasts bearing the homozygous Sigma-1R^E150K^ mutation do not display a significantly increased cell death compared to controls in basal condition, nor after treatment with common cell death-inducing insults. This indicates that the Sigma-1R^E150K^ variant may have a reduced cytotoxicity respect to other motor neuropathy-associated Sigma-1R variants [28, 31, 34]. This is in line with the relatively later onset and milder progressing clinical phenotype of the Italian patients compared to the other reported cases [17, 28]. Nevertheless, we found that inhibitors of the proteasome activity and autophagy flux significantly impair survival of homozygous Sigma-1R^E150K^ fibroblasts, indicating a crucial role of these two proteostatic systems in the maintenance of dHMN patient cell viability. This supports the notion that the balance between protein synthesis and degradation is crucial for neuronal cell homeostasis, function, and eventually survival. Alterations of this delicate balance caused by Sigma-1R mutation may be key triggers for the establishment of motor neuron dysfunction.

Our hypothesis is that the reduction of Sigma-1R function, either due to its decreased expression or its loss-of-function mutations or both (as in the case of dHMN patients), may cause chronic proteostatic stress culminating in enhanced sensitivity to cytotoxic insults targeting protein homeostasis. The disruption of the equilibrium between protein synthesis, quality control and degradation in Sigma-1R mutant cells may provoke irreversible cell damage and finally conduct to death of susceptible cells, such as neurons. Neuronal cell death is indeed the hallmark of most human neurodegenerative diseases including motor neuropathies.

**Fig. 6:**
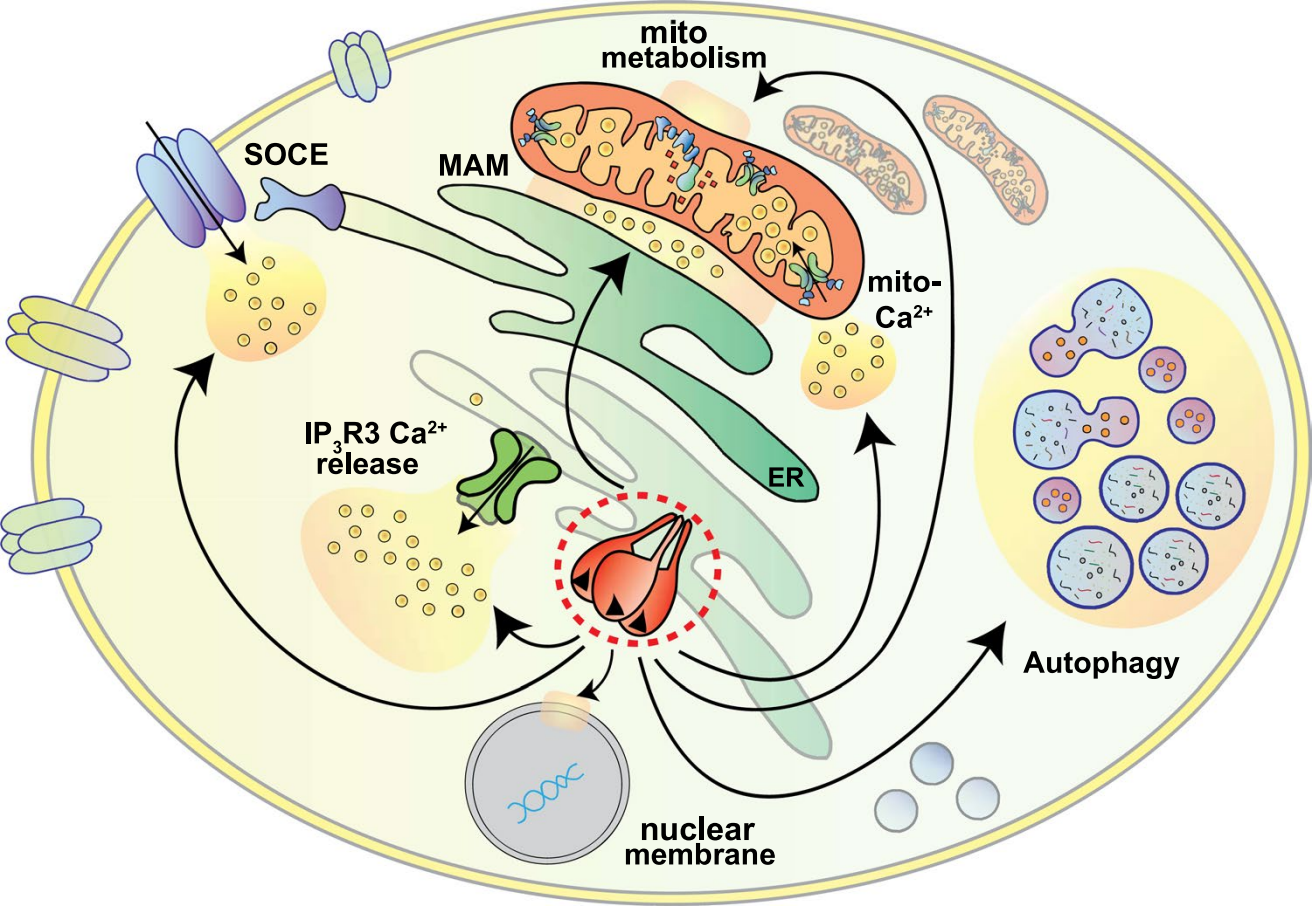
The pleiotropic roles of Sigma-1R protein in the control of cell homeostasis and metabolism. Schematic representation of the role of Sigma-1R in the regulation of multiple aspects of cell homeostasis and metabolism (intracellular Ca^2+^ dynamics, ER-mitochondria contacts, Ca^2+^ entry from the extracellular environment, ER protein stabilization, mitochondrial metabolism, autophagy). *ER* endoplasmic reticulum, *IP*_*3*_*R3* inositol 1,4,5-trisphosphate receptor type 3, *MAM* mitochondria-associated membranes; *mito-Ca*^*2*+^ mitochondrial Ca^2+^, *SOCE* store-operated Ca^2+^ entry

In addition, our data show that Sigma-1R has an additional crucial role in cell homeostasis as a modulator of global intracellular Ca^2+^ dynamics, regulating both the Ca^2+^ release from the ER stores and the Ca^2+^ entry from the extracellular milieu in patient cells (Fig. 6), as it was also reported in other cell systems [7, 60–63]. This feature is of major relevance for the physiology of excitable cells, such as neurons.

Indeed, in conditions of ER stress and ER Ca^2+^ depletion, Sigma-1R has been demonstrated to bind the ER Ca^2+^ sensor Stim1 and to translocate together with Stim1 to the plasma membrane, and this also happens following Sigma-1R agonist stimulation [7, 60]. Once at the cell membrane, the Sigma-1R-Stim1 complex interacts with and negatively regulates the Orai1 Ca^2+^ channel, thus dampening the Ca^2+^ influx through the plasma membrane. Sigma-1R thus acts as inhibitor of the so-called store operated Ca^2+^ entry (SOCE). In this way, by modulating the ER Ca^2+^ release and the extracellular Ca^2+^ entry, Sigma-1R is a pivotal regulator of the cytosolic Ca^2+^ response after agonist stimulation. As a consequence, the downregulation of Sigma-1R activity (by targeted RNAi and antagonist addition) leads to a decreased IP_3_R-mediated Ca^2+^ release and an enhanced SOCE amplitude; vice versa, Sigma-1R overexpression and Sigma-1R agonists stimulate intracellular Ca^2+^ release from the ER and decreases SOCE [7, 59, 60], despite a report showed that Sigma-1R forced expression augments both ER Ca^2+^ release and SOCE in MCF-7 cells [31]. Our results support the concept that Sigma-1R inhibits SOCE, as confirmed by our data from Sigma-1R overexpression in HEK-293T cells. Despite that, we observed an unpredicted reduction of SOCE in homozygous Sigma-1R^E150K^ cells that we can explain by a possible defective activity of the mutant protein on the Stim1-Orai1 complex. This may be due to an impaired binding of mutant Sigma-1R^E150K^ to Stim1, to a limited translocation of the Stim1-Sigma-1R complex to the membrane, or to a defective activation of the Orai1 channel. In line with this, recent findings pointed to a negative effect of Sigma-1R mutation on its binding partners, as shown for Sigma-1R^E102Q^ binding to the NMDA receptor [63]. However, future investigations will clarify whether Sigma-1R^E150K^ effectively acts according to these hypotheses.

In addition, we showed here that Sigma-1R^E150K^ significantly blunts the IP_3_-agonist induced cytosolic Ca^2+^ transient in patient fibroblasts by a combination of: (i) reduced ER Ca^2+^ release and (ii) impaired Ca^2+^ influx though plasma membrane. Since Sigma-1R binds the IP_3_R channel at MAMs leading to the stabilization of the IP_3_R and stimulation of its Ca^2+^channel activity [60], we hypothesize that the decreased ER Ca^2+^ release is primarily due to an impairment of IP_3_R function, as suggested by the reduced IP_3_R3 protein level observed in patient cells. In addition to this, the E150K mutation may disrupt Sigma-1R function as IP_3_R-chaperone thus negatively affecting the Ca^2+^ release activity of the channel. Nevertheless, this hypothesis needs to be experimentally tested.

Interestingly, our data also evidenced the important contribution of Sigma-1R in the maintenance of cellular energy homeostasis. Indeed, the homozygous Sigma-1R^E150K^ mutation dramatically affects mitochondrial respiration and ATP production in patient cells. This is of paramount importance for high energy demanding cells as motor neurons, which are intrinsically extremely sensitive to insults tackling mitochondrial metabolism, Ca^2+^ dysregulation and oxidative stress [64–66]. In the same line, Sigma-1R knock-down or inhibition by antagonists has been shown to impair the global Ca^2+^ signalling and mitochondrial energetics during neuroblastoma cell line ER stress, thus modulating mitochondrial Ca^2+^ uptake and the consequent energy fuelling during early UPR response [67], which may dramatically impact on the ability of cell to respond to stress and finally undermine cell survival.

Concluding, our study provided a significant advancement in the comprehension of the molecular mechanisms leading to Sigma-1R-linked recessive dHMN revealing unexplored roles of the receptor pathogenic variants in the inter-organelle communication, global cellular Ca^2+^ dynamics and mitochondrial function, and thus assigning to Sigma-1R a key position in the control of cell homeostasis and energy metabolism (Fig. 6).

Moreover, the relevance of these findings can be translated to a variety of human pathologies, especially those involving mitochondria dysfunction and ER stress, such as Alzheimer’s disease and other neurodegenerative disorders, and thus contributing to pave new therapeutic roads for the development of tailored interventions against these still unmet human pathologies.

## Materials and methods

### Cell cultures, treatments and transfection

All experiments were performed on primary skin fibroblasts derived from two sibling patients carrying the p.E150K Sigma-1R point mutation or control fibroblast cultures, which were obtained following the receipt of informed consent from all participants or as previously described [68]. The study was conducted in accordance with the Declaration of Helsinki, ensuring compliance with the ethical standards and protocols established at the San Camillo Forlanini Hospital in Rome, Italy, Unit of Neurology and Neurophysiopathology. HEK-293T cells were used to perform over-expression experiments. Primary fibroblasts and HEK-293T were cultured in Dulbecco’s modified Eagle’s medium (DMEM) (ThermoFisher Scientific, Waltham, Massachusetts, US), supplemented with 20% or 10% Fetal Bovine Serum (FBS) respectively (ThermoFisher Scientific, Waltham, Massachusetts, US), containing penicillin (100U/ml) and streptomycin (100 μg/ml) (ThermoFisher Scientific, Waltham, Massachusetts, US). Cells were maintained in culture at 37 °C, with 5% CO_2_. Lipofectamine 2000 (ThermoFisher Scientific, Waltham, Massachusetts, US) were used to transfect cells according to the manufacturer’s instruction. The plasmids used to express Flag-tagged WT or E150K-mutant Sigma-1R and the split-GFP based SPLICS probe were described elsewhere [30, 43].

Cell treatments with chemical compounds were performed in the culture medium: chloroquine was dissolved in water, MG-132 was dissolved in DMSO (Merck, Darmstadt, Germany). Cells were treated with equal amounts of the inhibitor solvent as vehicle control. For serum-deprivation experiments (Starvation condition), fibroblasts were washed with phosphate-buffered saline (PBS) (Merck, Darmstadt, Germany) and then were incubated for 4 h in sterile modified Krebs–Ringer buffer (KRB: 135 mM NaCl, 5 mM KCl, 1 mM MgSO_4_, 0.4 mM K2HPO_4_, 20 mM HEPES, pH = 7.4, supplemented with 1 mM CaCl_2_).

### Immunofluorescence analysis

Control or patients’ fibroblasts were seeded on 13 mm glass coverslips. The day after, cells were fixed with PFA 4% (Santa Cruz Biotecnology, US) for 20 min in the dark, permeabilized for 10 min in PBS containing 0.2% triton X-100 (Merck, Darmstadt, Germany), and then blocked in PBS containing 5% BSA/0.2% triton X-100 for 1 h. Then, cells were incubated with the following primary antibodies overnight at 4 ℃: anti mouse-TOM20 1:200 (SantaCruz Biotecnology, US, Cat: sc-17764), anti-mouse calreticulin 1:100 (BD Bioscience Cat: 612127), anti-rabbit calreticulin (ThermoFisher Scientific, Waltham, Massachusetts, US, Cat:PA3-900) anti-rabbit Sigma-1R 1:100 (Merck, Darmstadt, Germany, Cat: HPA018002), anti-rabbit LC3B 1:100 (Cell Signaling Technology, Danvers, Massachusetts, US), anti-rabbit p62/SQSTM1 (Merck, Darmstadt, Germany, Cat: P0067), anti LAMP2/CD107b (Abcam, Cambridge, United Kingdom, Cat: ab25631). The appropriate AlexaFluor-conjugated secondary antibodies (AlexaFluor-488 or −555, Life Technologies, Carlsbad, California, US) were incubated 1:500 in PBS containing 5% BSA, in the dark at room temperature and the coverslips were mounted with Mowiol reagent (Merck, Darmstadt, Germany). Images were taken with a Leica TCS-SP5-II confocal microscope equipped with a 100 × / 1.4 N.A. plan-apochromat objective and were processed using LAS AF (Leica Microsystems GmbH, Wetzlar, Germany) software.

For colocalization measurements, images were analyzed using the Fiji image processing package based on ImageJ [69]. Colocalization index were quantified by Manders’ coefficient using JACoP plugin for ImageJ after iterative deconvolution process, background subtraction and conversion of images to 8-bit type. Any further manipulation has been avoided. For the analysis of the number of p62 and LC3-positive dots, after the immunostaining with antibodies against endogenous SQSTM1/p62 and LC3 proteins, “3D Object counter” function of the ImageJ-based Fiji image processing package [69] was used. The threshold was manually set to remove objects with a volume below 2 or above 1000 voxels in order to reduce noise and avoid aggregation artifacts, respectively. On each image, the resulting number of puncta was divided for the number of cells in each field.

### ER-mitochondria contact sites

For the assessment of ER-mitochondria contact sites, a split-GFP-based sensor was implemented as described in Calì and Brini [70]. Briefly, fibroblasts were seeded on 13 mm glass coverslips and the day after cells were transfected with equimolar concentrations of the GFP_1–10_ fragment, targeted to the outer mitochondrial membrane and the ER-targeted β_11_ strand. After 24 h, live confocal images of the reconstituted GFP fluorescent signal were acquired, and then analyzed using the “3D Object counter” function of Fiji, an image processing package based on ImageJ [69]. The threshold was set manually and objects with a volume below 5 or above 500 voxels were removed in order to eliminate noise and aggregation artifacts.

### Western blot

Primary fibroblasts were lysed in cold RIPA buffer containing 125 mM NaCl, 25 mM Tris–Cl (pH 7.4), 1 mM EGTA-Tris (pH 7.4), 1% Triton X-100, 0.5% sodium deoxycholate, 0.1% SDS, and Complete EDTA-free protease inhibitor cocktail (Roche, Basel, Switzerland) for 30 min on ice. Extracts were cleared by centrifugation for 15 min at 14.000 g. Cellular lysates were then collected, and protein concentration was determined by BCA method (ThermoFisher Scientific, Waltham, Massachusetts, US). Proteins (20 μg) were subjected to 4–12% gradient SDS-PAGE, blotted on Immobilon-P membranes (Merck, Darmstadt, Germany), incubated with the indicated antibodies, and revealed by Alliance Q9 Advanced chemiluminescent detection system (UVITEC, Cambridge, UK). Immuno-stained bands were quantified using Fiji image processing package based on ImageJ [69] without any manipulation.

### Cell viability assay

Cell viability assay was performed as described in Gregianin et al. [30]. Briefly, fibroblasts were seeded on 96-well plate (8 × 10^3^ cells/well) and the day after cells were left untreated (Basal) or treated with the indicated compounds for 24 h. One hour before the assay, cells were incubated with MTS (3-(4,5-dimethylthiazol-2yl)−5-(3-carboxymethoxyphenyl)−2-(4-sulfophenyl)−2Htetrazolium) salt solution (CellTiter 96^®^ AQueous One Solution; Promega, Madison, Wisconsin, US). Plates were incubated at 37 ℃ for 1 h in the dark and read at λ = 490 nm in a Multiskan EX multiplate reader (ThermoFisher Scientific, Waltham, Massachusetts, US). The percentage of viable cells was calculated as a ratio of optical density of treated versus untreated cells.

### RNA extraction, reverse transcription, and quantitative real-time PCR

Total RNA was isolated from control and patient fibroblasts using Trizol Reagent (Thermo Fisher Scientific, Waltham, Massachusetts, US) following the manufacturer’s procedures. cDNA was generated from 500 ng RNA with SuperScriptII Reverse Transcriptase (Thermo Fisher Scientific, Waltham, Massachusetts, US), and real-time PCR was performed using iQ SYBR Green Supermix and the iQ5iCycler (Bio-Rad, Hercules, California, US). The oligonucleotides used for amplification are:

ATF4: forward, 5’-GTTCTCCAGCGACAAGGCTA-3’; reverse, 5’-ATCCTGCTTGCTGTTGTTGG-3’;

sXBP: forward, 5’-CTGAGTCCGAATCAGGTGCAG-3’; reverse, 5’-ATCCATGGGGAGATGTTCTGG-3’;

total XBP: forward, 5’-TGGCCGGGTCTGCTGAGTCCG-3’; reverse, 5’-ATCCATGGGGAGATGTTCTGG-3’;

EDEM: forward, 5’-CAAGTGTGGGTACGCCACG-3’; reverse, 5’-AAAGAAGCTCTCCATCCGGTC-3’;

MCU: forward, 5’-GCAGAATTTGGGAGCTGTTT-3’; reverse, 5’-GTCAATTCCCCGATCCTCTT-3’;

MICU1: forward, 5’-TATACTCCGGTCCCATAGGC-3’; reverse, 5’-ATGCTCTTGTCGGGGTACAG-3’;

MICU2: 5’-GGCAGTTTTACAGTCTCCGC-3’; reverse, 5’-AAGAGGAAGTCTCGTGGTGTC-3’;

RLP32: forward 5’-CATCTCCTTCTCGGCATCA-3’; reverse, 5’-CTGGGTTTCCGCCAGTTAC-3’.

### Subcellular fractionation

About 1 × 10^6^ primary skin fibroblasts were harvested with trypsin–EDTA (Merck, Darmstadt, Germany) and centrifuged at 800 g for 5 min at 4 ℃. Then, cells were washed twice with cold PBS and the pellet were resuspended in 2 ml of ice-cold isotonic cell lysis buffer containing protease and phosphatase inhibitors. Cells were gently lysed by 80 strokes in a Dounce homogenizer on ice and subsequently the homogenate centrifuged at 900 g for 10 min at 4 ℃ to collect nuclear fraction. The supernatant containing cytoplasm, membranes and mitochondria was transferred in a fresh tube and centrifuged at 7500 g for 15 min at 4 ℃ to collect mitochondria. Finally, the supernatant was ultra-centrifuged at 100,000 g for 1 h at 4 ℃ to separate the membrane fraction.

### Transmission electron microscopy

Fibroblasts were grown in 24-well plates and fixed with 2.5% glutaraldehyde in 0.1 M sodium cacodylate buffer pH 7.4 for 1 h at 4 ℃, post-fixed with a mixture of 1% osmium tetroxide and 1% potassium ferrocyanide in 0.1 M sodium cacodylate buffer for 1 h at 4 ℃ and incubated overnight in 0.25% uranyl acetate at 4 ℃. After three water washes, samples were dehydrated in a graded ethanol series and embedded in an epoxy resin (Sigma-Aldrich, St. Louis, Missouri, US). Ultrathin sections (60–70 nm) were obtained with an Ultrotome V (LKB, Gungahlin, Australia) ultramicrotome, counterstained with uranyl acetate and lead citrate, and viewed with a Tecnai G2 (FEI, Hillsboro, Oregon, US) transmission electron microscope operating at 100 kV. Images were captured with a Veleta digital camera (Olympus Soft Imaging System, Olympus, Shinjuku City, Tokyo, Japan).

### Calcium measurements

*Cytosolic Ca*^*2*+^ *measurements*. The experimental procedure was performed as reported in Gregianin et al. [30]. Briefly, fibroblasts were seed at 80% confluence in the 13 mm coverslip the day before the experiment in DMEM 20% FBS. The day after, cells were loaded with 2 μM Fura-2-AM (Life Technologies, Carlsbad, California, US) in KRB for 20 min at 37 ℃ and then washed with KRB for other 20 min. Images were acquired every 1 s with a Zeiss Axiovert 200 microscope equipped with a PlanFluar 60 × / 1.3 N.A. oil immersion objective (Zeiss) and a high-sensitivity 16-bit Evolve 512 Delta EMCCD (Teledyne Photometrics, Tucson, Arizona, US) camera. Images were collected by alternatively exciting the fluorophore at 340 and 380 nm and fluorescence emission recorded through a 515/30 nm band-pass filter (Semrock®, Rochester, New York, US). Changes in fluorescence were expressed as ΔR/R0, where ΔR = (R – R0), R is the 340/380 nm ratio at time t and R0 is the 340/380 nm ratio at the beginning of the experiment, before agonist addition.

*SOCE activity measurements*. The experimental procedure was performed as reported in [71]. Briefly, fibroblasts or HEK-293T cells were seeded at 80% of confluence on 13 mm glass coverslips. The day after, cells were incubated with 2 μM Fura-2-AM (Life Technologies, Carlsbad, California, US) in KRB for 20 min at 37 °C and then washed twice with KRB containing 200 μM EGTA to chelate extracellular Ca^2+^. The experiments were performed in KRB containing EGTA, and CPA was added to induce ER Ca^2+^ depletion. Finally, a 2 mM Ca^2+^ solution was added to induce Ca^2+^ influx across the cell membrane.

*Mitochondrial Ca*^*2*+^ *measurements.* The experimental procedure was performed as reported in [30]. Briefly, mitochondrial Ca^2+^ measurements were performed in human fibroblasts after 48 h infection with the mitochondrial-targeted GCaMP6f (4mt-GCaMP6f) adenovirus using a Leica TCS-SP5-II confocal microscope equipped with a 40 × / 1.3 N.A. plan-apochromat objective and were processed using LAS AF (Leica Microsystems GmbH, Wetzlar, Germany) software. 4mt-GCaMP6f was alternately excited at 474 and 410 nm and images were collected through a 535/20 band- filter (Semrock®, Rochester, New York, US) [72]. For all the experiments, exposure time was set to 200 ms and images were acquired every 2 s. Changes in mitochondrial Ca^2+^ levels were expressed as ΔR/R0, where ΔR = (R – R0), R is the 474/410 nm fluorescence ratio at time t and R0 is the ratio at the beginning of the experiment, before agonist addition. Analysis was performed using the Fiji image processing package based on ImageJ [69]. Images were background corrected frame by frame by subtracting the mean pixel intensity value of a cell-free region of interest.

### Oxygen consumption and extracellular acidification measurements

Oxygen utilization and glycolytic activity were assessed by measuring the Oxygen Consumption Rate (OCR) and Extra Cellular Acidification Rate (ECAR) of control and patient fibroblasts with the Seahorse XF24 extracellular flux analyzer (Agilent, Santa Clara, California, US), as previously described [73]. Cells were plated on XF24 cell culture plates (2 × 10^4^ cell/well) and the day after the growth medium was replaced with unbuffered DMEM (Merck, Darmstadt, Germany) and equilibrated for 1 h at 37 °C. OCR and ECAR values were normalized to the protein content of each sample.

### ROS measurements

Primary fibroblasts (8 × 10^3^) were seeded on 96-well plates and ROS levels were detected using the 5-(and-6)-chloromethyl-2′,7′-dichlorodihydrofluorescein diacetate, acetyl ester (CM-H2DCFDA, 1 mM; Molecular Probes, Eugene, Oregon, US) dissolved in DMEM media without phenol red and FBS. Fluorescence (Excitation λ = 485 nm; Emission λ = 538 nm) was recorded using a Fluoroskan Ascent FL plate reader (ThermoFisher Scientific, Waltham, Massachusetts, US).

## Supplementary Information

Below is the link to the electronic supplementary material.

Supplementary file1 (PDF 1107 KB)

## Data Availability

The data supporting the findings of this study are found in the article and the supplementary material. The corresponding author will make all relevant raw data available upon reasonable request.
