# Supplementary material for: Mutated sigma-1R disrupts cell homeostasis in dHMN patient cells
Source: Cell Mol Life Sci. 2025 Apr 9;82(1):151. doi: 10.1007/s00018-025-05676-y (PMC11981993; doi:10.1007/s00018-025-05676-y)
Supplement: Supplementary file 1 — Supplementary file1 (PDF 1107 KB) [file 18_2025_5676_MOESM1_ESM.pdf]

Supplementary Material Zanin et al.

Title:

Mutated Sigma-1R disrupts cell homeostasis in dHMN patient cells

Authors:

Sofia Zanin [1], Francesco Ciscato [2,3], Antonio Petrucci[4], Annalisa Botta[5], Federico Chiossi[6], Giovanni Vazza [7]*, Rosario Rizzuto [3]*, Giorgia Pallafacchina [2,3]*

Affiliations:

[1] Laboratory for Genetics of Mitochondrial Disorders, UMR 1163, Université de Paris, Institut Imagine, Paris, France.

[2]Neuroscience Institute, Italian National Research Council CNR, Padua, Italy.

[3]Department of Biomedical Sciences, University of Padua, Padua, Italy

[4]Center for Neuromuscular and Neurological Rare Diseases, S. Camillo Forlanini Hospital, Rome, Italy.

[5]Medical Genetics Section, Department of Biomedicine and Prevention, University of Rome Tor Vergata, Rome, Italy.

[6]Chimie ParisTech, PSL University, CNRS, Institut de Recherche de Chimie, Paris, France.

[7]Department of Biology, University of Padua, Padua, Italy

*Corresponding authors: G.P. giorgia.pallafacchina@unipd.it, R.R. rosario.rizzuto@unipd.it and G.V. giovanni.vazza@unipd.it.

Supplementary Figure S1

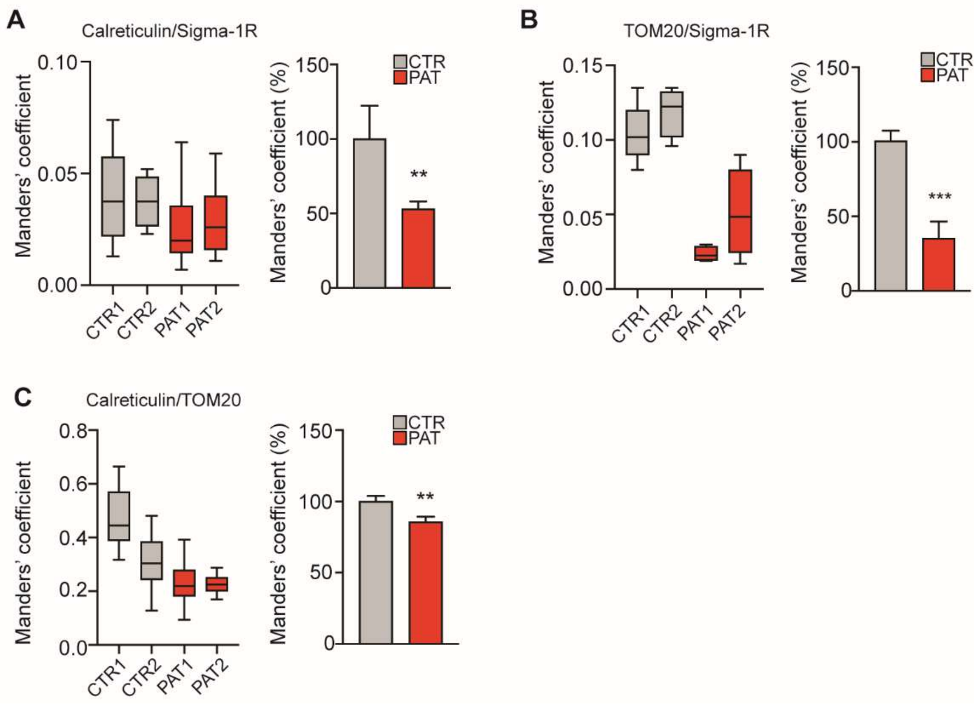

Supplementary Fig.S1 Altered distribution of Sigma-1R protein and impaired ER-mitochondria contacts in dHMN patient fibroblasts. A, B The graphs represent the colocalization of the ER calreticulin (A) or mitochondrial TOM20 (B) proteins to Sigma-1R expressed as Manders' coefficient calculated for each individual (left boxplot) or as average of the percentage respect to controls (right histogram) and relative to the images in main Fig. 1 A-B. **C** The graphs represent the colocalization of calreticulin (in red) to TOM20 (in green) protein expressed as Manders' coefficient calculated for each individual (left boxplot) or as average of the percentage respect to controls (right histogram) and relative to the immunostaining in main Fig. 1 E. Data information: box plot data are from at least ten confocal images and three independent experiments. Student's t-test was used for statistical analysis. Data are reported as mean ± SEM. **P < 0.01 ***P < 0.001

Supplementary Figure S2

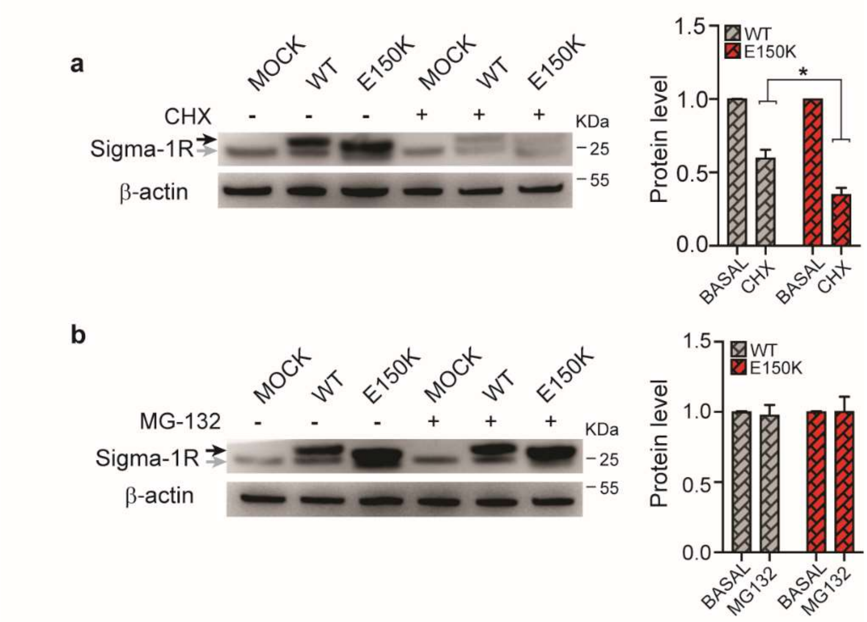

Supplementary Fig.S2 dHMN patient fibroblasts have reduced Sigma-1R protein level. A, B Representative immunoblotting and relative quantification of Sigma-1R protein level from lysates of HEK-293T cells transfected with empty (MOCK), wt Flag-Sigma-1R or Flag-Sigma-1R^{E150K} constructs for 24 h. Cells were treated with vehicle (BASAL), 5μM cycloheximide (CHX) for 24 h (A) or 1μM MG-132 for 8h (B). β-actin is used as loading control. The two Sigma-1R bands correspond to the endogenous (grey arrow) and the Flag-tagged overexpressed (black arrow) protein. Data information: data in A-B are from three independent experiments. Data are presented as mean ± SEM. *$p < 0.05$

Supplementary Figure S3

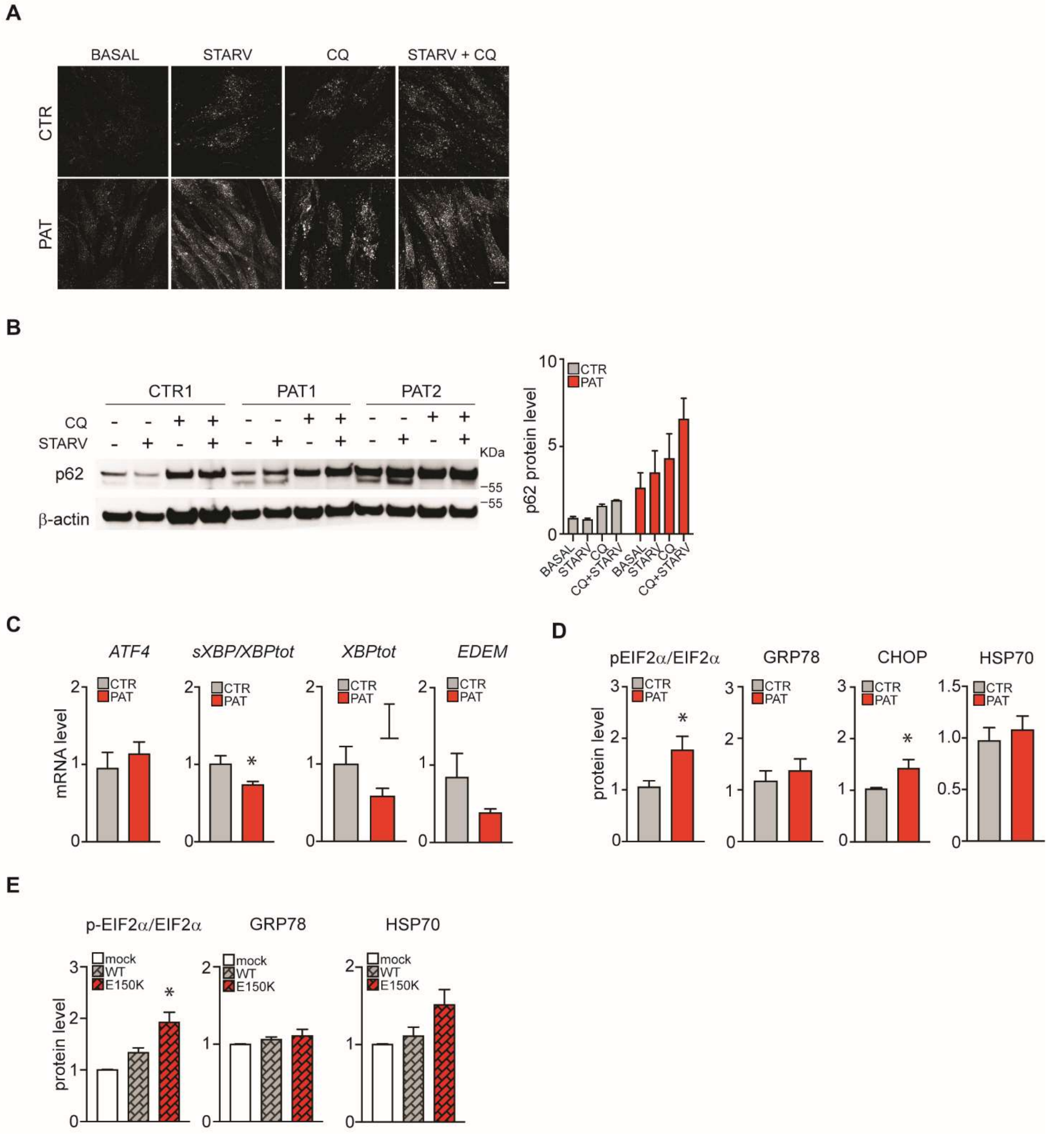

Supplementary Fig.S3 Homozygous Sigma-1R^{E150K} fibroblasts display enhanced autophagy and increased level of ER stress markers. A Representative confocal images of LC3B immunostaining in control (CTR) and patient (PAT) fibroblasts in standard conditions or after starvation for 4 h (STARV), with or without chloroquine (CQ, 50 μM for 1 h) relative to main Fig. 3 B. **B** Representative immunoblotting and relative quantification of p62 level in control (CTR) and patient (PAT1, PAT2) fibroblasts in standard conditions or after starvation in PBS for 4 h (STARV), with or without chloroquine (CQ, 50 μM for 1 h). β-actin was used as loading control. **C** Real-time PCR quantification of the mRNA level and immunoblotting band quantification of the protein level of the indicated ER-stress markers. Each bar represents the mean value of at least 3 measures from two individuals and relative to control. **D, E** Quantification of the protein level of the indicated ER-stress markers relative to the immunoblot bands of main Fig. 3 E, F. Data information: The results are representative of at least two (panels A and B), three (panel C), five (panels D and E) independent experiments. Data of the graphs reported in A, C and D are from at least 10 confocal images. Data are presented as mean ± SEM. * $p < 0.05$. Scale bar = 10 μm

Supplementary Figure S4

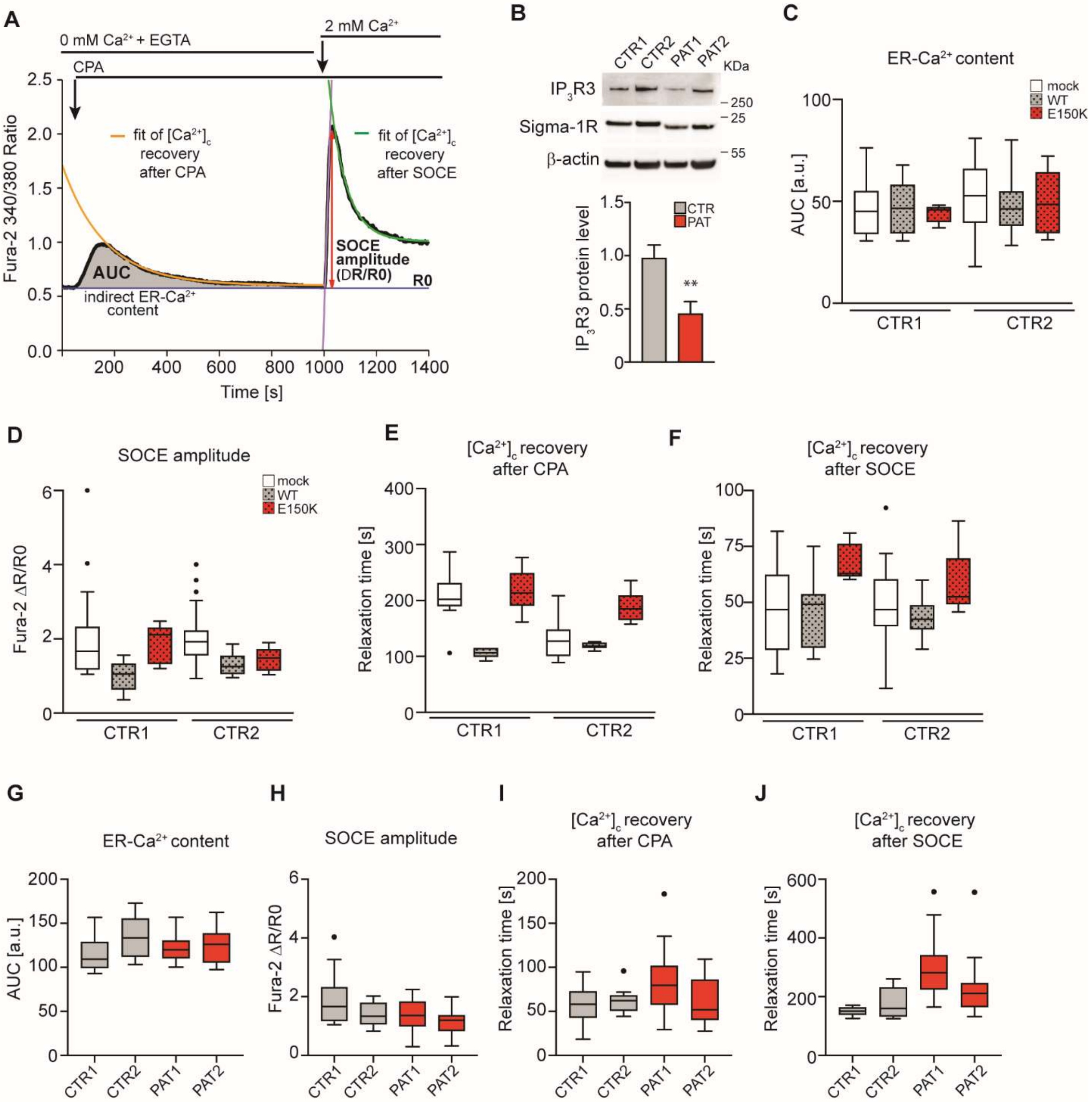

Supplementary Fig.S4 Assessment of the intracellular Ca^{2+} dynamics in Sigma-1R^{E150K} expressing fibroblasts. A Schematic representation of the parameters calculated by the analysis of the cytosolic Ca^{2+} traces obtained with Fura-2AM shown in main Fig. 4. The peak after the addition of the SERCA pump inhibitor CPA was calculated as Fura-2AM $\Delta R/R0$ ratio and represents the ER Ca^{2+} release. The area under the curve (AUC), calculated in an interval of 20 min, represents the indirect measure of the ER-Ca^{2+} content. The peak after the addition of 2 mM Ca^{2+} represents the extracellular Ca^{2+} influx (SOCE) and was calculated as Fura-2AM $\Delta R/R0$ ratio. The cytosolic Ca^{2+} recovery rate is estimated by the relaxion time (τ : the time to reach the 37% of the peak value) extrapolated from the equation of the exponential fit of the Ca^{2+} curve decrease after CPA (orange) and after SOCE (green) as described in Materials & Methods. The R0 curve represents the mean basal value of the Fura-2 340/380 fluorescence ratio. **B** Representative immunoblotting and relative quantification of IP_3R3 protein level in control (CTR1, CTR2) and patient (PAT1, PAT2) fibroblasts. **C-F** Box plots represent the Fura-2AM $\Delta R/R0$ values used to calculate the percentages of main Fig. 4 E-H, for each condition and for the two cell lines used (CTR1-CTR2). **G-J** Box plots represent the Fura-2AM $\Delta R/R0$ values used to calculate the percentages of main Fig. 4 J-M measured for control (CTR1,CTR2) and patient (PAT1,PAT2) fibroblasts. Data information: Data in C-F are from three independent experiments. Data in G-J are from five independent experiments. Data are shown as the means ± SEM. Student's t-test was used for statistical significance assessment. **P < 0.01. Black circles represent the outliers

Supplementary Figure S5

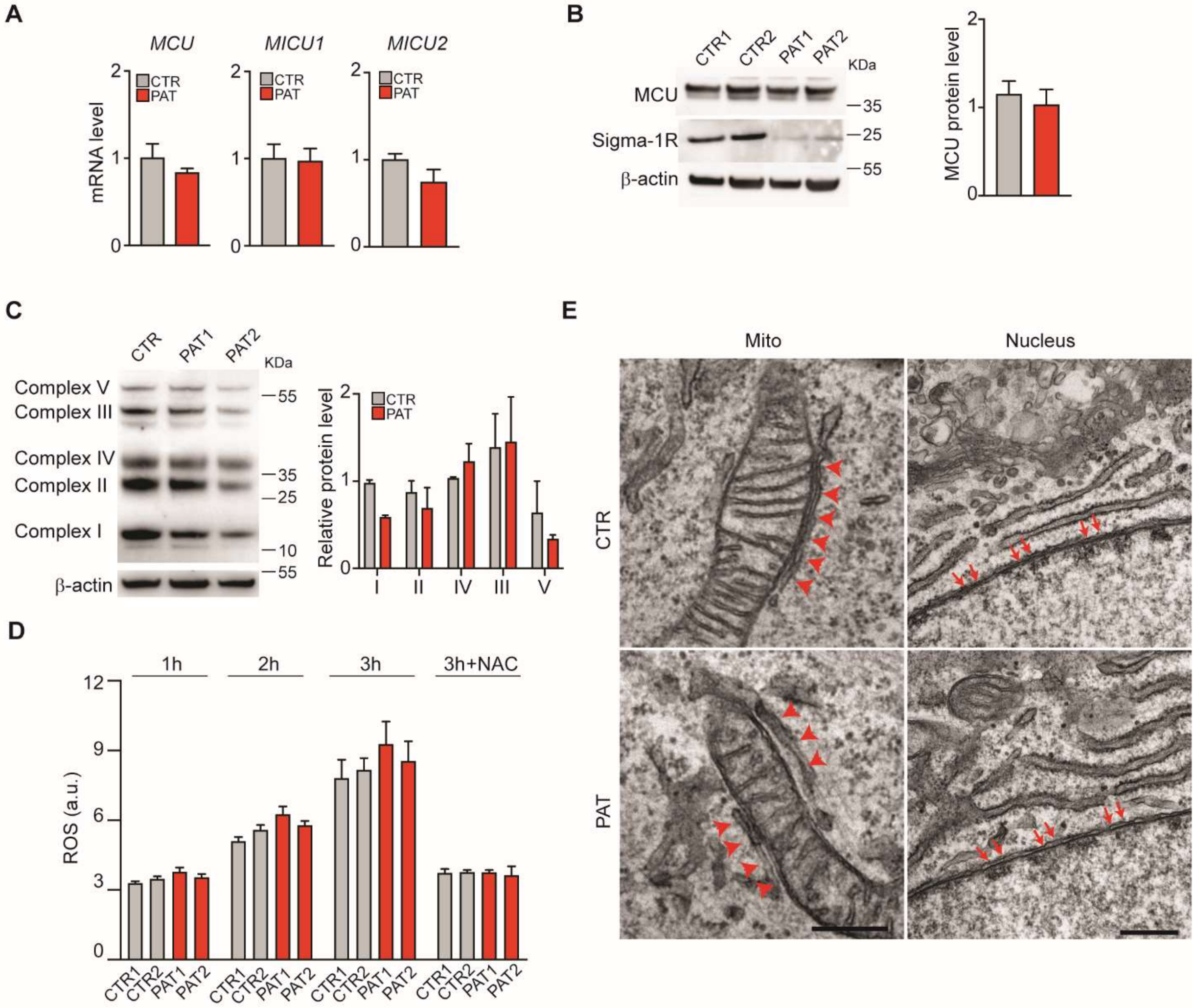

Supplementary Fig.S5 Assessment of mitochondrial complexes' expression and mitochondria ultrastructure in dHMN patient fibroblasts. A Real-time PCR quantification of the mRNA level of the indicated MCU components. Bars represent the mean value of samples from two patients (PAT) and two control individuals (CTR), relative to controls. **B** Representative immunoblotting and quantification of the MCU protein level in control (CTR1, CTR2) and patient (PAT1, PAT2) fibroblasts. Sigma-1R band is also shown and β-actin is used as loading control. **C** Representative immunoblotting and relative quantification of OXPHOS components' protein levels in control (CTR) and patient (PAT1, PAT2) fibroblasts. β-actin is used as loading control. **D** Quantification of ROS levels in control and patient fibroblasts by measuring 2',7'-dichlorofluorescein fluorescence. The antioxidant agent N-acetyl-cysteine (NAC) was pre-incubated 30 min to block ROS production. **E** Representative transmission electron microscopy images depicting the mitochondria-ER contacts (left) and rough ER with nuclear envelope (right) of control (CTR) and patient (PAT) fibroblasts. Arrowheads indicate the ER-mitochondria contact sites. Arrows indicate the nuclear membrane envelope. Data information: Data in A, C-E are from 3 independent experiments. Data in B is from five independent experiments. Data are reported as mean ± SEM values. Scale bar = 500 nm
